# Supplementary material for: Tetris-inspired detector with neural network for radiation mapping
Source: Nat Commun. 2024 Apr 9;15:3061. doi: 10.1038/s41467-024-47338-w (PMC11004156; doi:10.1038/s41467-024-47338-w)
Supplement: Supplementary file 1 — Supplementary Information [file 41467_2024_47338_MOESM1_ESM.pdf]

Tetris-inspired detector with neural network for radiation mapping: Supplementary Information

Ryotaro Okabe[1,2,*,†], Shangjie Xue[1,3,4,*], Jayson R Vavrek[5,*], Jiankai Yu[3], Ryan Pavlovsky[5], Victor Negut[5], Brian J Quiter[5], Joshua W Cates[5], Tongtong Liu[1,6], Benoit Forget[3], Stefanie Jegelka[4], Gordon Kohse[7], Lin-wen Hu[7,†], and Mingda Li[1,3,†]

[1]Quantum Measurement Group, Massachusetts Institute of Technology, Cambridge, MA 02139, USA
[2]Department of Chemistry, Massachusetts Institute of Technology, Cambridge, MA 02139, USA
[3]Department of Nuclear Science and Engineering, Massachusetts Institute of Technology, Cambridge, MA 02139, USA
[4]Department of Electrical Engineering and Computer Science, Massachusetts Institute of Technology, Cambridge, MA 02139, USA
[5]Applied Nuclear Physics Program, Lawrence Berkeley National Laboratory, Berkeley, CA 94720, USA
[6]Department of Physics, Massachusetts Institute of Technology, Cambridge, MA 02139, USA
[7]Nuclear Reactor Laboratory, Massachusetts Institute of Technology, Cambridge, MA 02139, USA
[*]These authors contributed equally to this work
[†]Corresponding authors: rokabe@mit.edu, lwhu@mit.edu, mingda@mit.edu

Contents

I Supplementary Note 1: Monte Carlo Simulation Setting and Data Representation 1
II Supplementary Note 2: Deep Neural Network Architecture 4
III Supplementary Note 3: Evaluation of Static Detector 4
III.1 Angular and Position Resolution . . . 4
III.2 Gamma Energy Dependency . . . 6
III.3 Directional Prediction of Two Radiation Sources with Single Filter Layer Model . . . 8
III.4 Effect of Background Noise . . . 10
IV Supplementary Note 4: Radiation Source Mapping with Maximum A Posteriori (MAP) Estimation 12
V Supplementary Note 5: Mapping Two Radiation Sources 17
VI Supplementary Note 6: Radiation Mapping with a Rotating Detector Moving along the Circular Trajectory. 19
VII Supplementary Note 7: Experimental Validation of Radiation Mapping with the 2×2 Square Detector 25
VIII Supplementary Note 8: Guidance for Running the Codes for Radiation Mapping 28

I Supplementary Note 1: Monte Carlo Simulation Setting and Data Representation

Our simulated radiation detectors consist of two materials: CdZnTe to absorb radiation and Pb as padding layers. The density of CdZnTe and Pb are set at 5.76 g/cm^3 and 11.35 g/cm^3, respectively. Table S1 shows the MC simulation settings to generate training data and the filter layers. Figure S1 shows the position of the radiation sources used for training of the S-shape detector. The 64 near-field filters of the S-shape detector are visualized in Fig. S2.

Table S1. The parameter setting for Monte-Carlo simulations for radiation source detection.

	Training, Testing Data	Filter
The number of photon particles	50000	80000
The number of angle sectors	64	64
radiation source energy [MeV]	0.5	0.5
The detector-source distance [cm]	20 ∼500	near: 50, far: 500
The number of simulation data	3000	64×2 channels

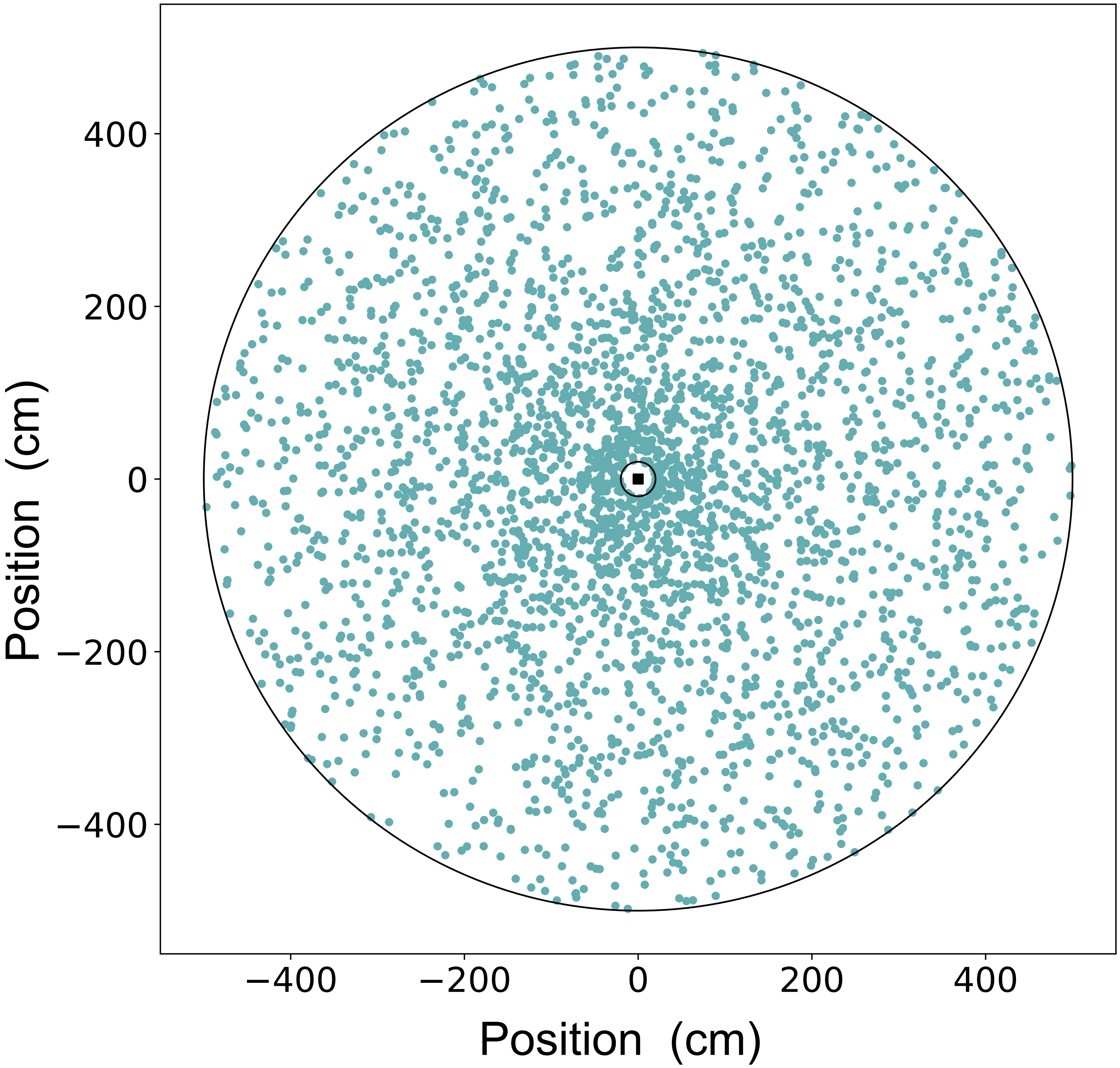

Figure S1. Visualizations of the radiation source positions of training data.
the black square is the detector's position. Blue dots are the radiation source positions (3,000 data). The circles show the minimum and maximum distance between the radiation sources and the detector.

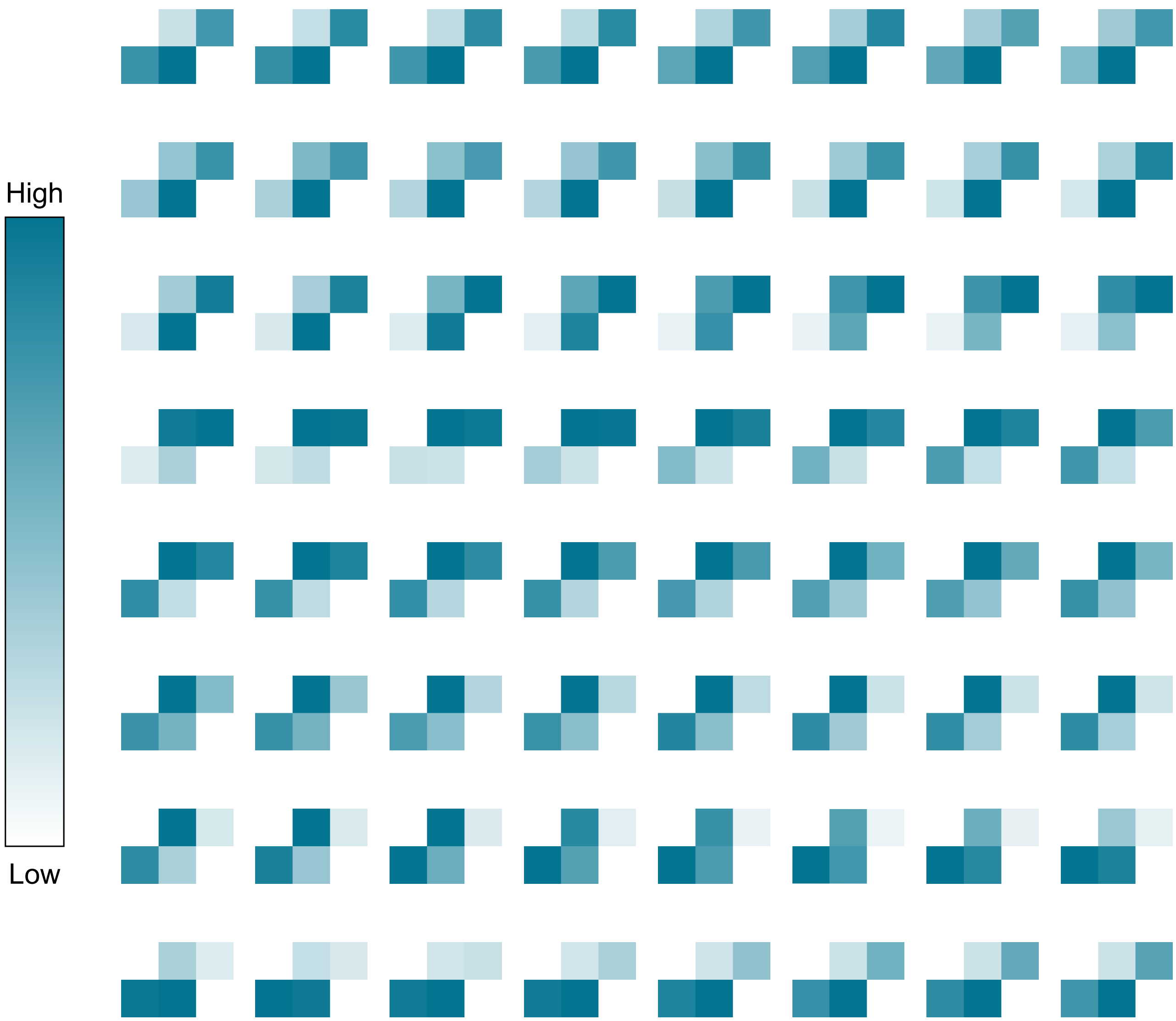

Figure S2. **The near-field global filters of the S-shape configuration.**
All of the 64 arrays represent the filters with a radiation source coming from a different direction. As the color bar of the left side shows, the intense color represents higher input signals. We use another set of 64 far-field filters to capture the direction of radiation sources.

II Supplementary Note 2: Deep Neural Network Architecture

We implemented a U-shaped neural network to predict the angular information from the detector's signal. The dimensions of each layer are shown in Fig. S3. The input data goes through 64 filter layers of both far- and near-field channels and is embedded as $(2,64)$ matrix. The U-Net is then applied to this embedded data and outputs an array of 64 elements. Table S2 summarizes the hyperparameters of the U-shaped neural network.

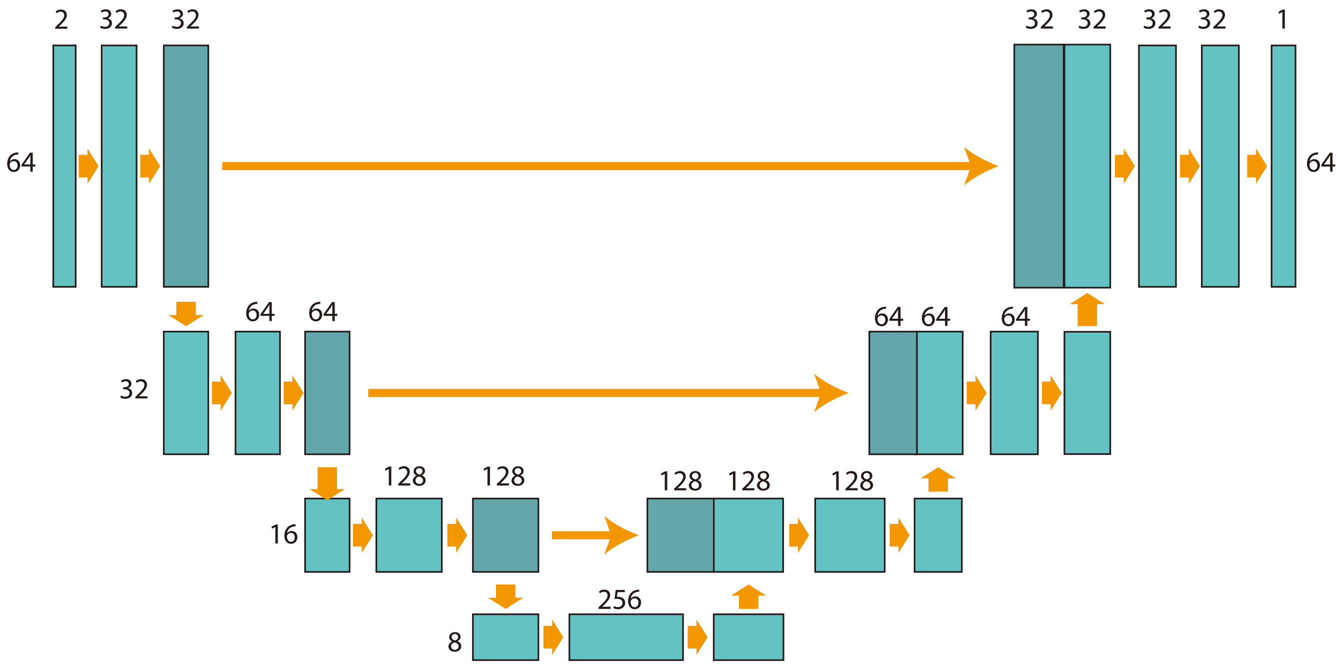

Figure S3. Model architecture of U-Net convolutional neural networks.
The number near the rectangular shapes represents the digits of matrices. The model consists of convolution, transposed convolution, max pooling and concatenation.

Table S2. The parameter setting of U-Net

Hyperparameter	1-source detector	2-source detector
Epochs	200	5000
The number of angle sectors	64	64
batch size	256	256
AdamW optimizer learning rate	1×10^{-3}	1×10^{-3}
AdamW optimizer weight decay coefficient	3×10^{-5}	3×10^{-5}
Training data size	2700	2700
Testing data	300	300

III Supplementary Note 3: Evaluation of Static Detector

III.1 Angular and Position Resolution

In our academic journal paper, we present a comprehensive analysis of the performance of various radiation detectors in predicting the direction of radiation sources. Table 1 provides a summary of the prediction errors associated with each detector. The data highlight the variations in accuracy across the detectors, which is crucial in selecting the most suitable detector for specific applications.

In Figure S4, we present a detailed visualization of the prediction errors for each detector, presented as a function of the source direction angle on a polar axis. This graphical representation offers valuable insights into the strengths and weaknesses of each detector at different angles. Understanding these variations allows us to optimize detector selection and placement, leading to more reliable radiation detection systems in real-world scenarios.

Among the detectors investigated, the square shape detector works as the standard. It demonstrates an exceptional ability to detect the direction of radiation sources evenly across the entire direction range. This uniform performance makes it a highly reliable choice for accurately identifying radiation sources from any direction. On the other hand, the Tetris-inspired detectors exhibit a more nuanced performance profile. These detectors show varying levels of accuracy depending on the angles of the radiation sources. Specifically, the S-shape detector excels in predicting radiation sources located in the top right and bottom left regions with smaller loss, while it faces challenges when dealing with sources from the top left and bottom right regions with higher loss. Similarly, the J-shape detector demonstrates proficiency in predicting radiation sources originating from the bottom right region. However, it encounters difficulties when attempting to predict the direction of radiation sources from the bottom and left region. In contrast, the T-shape detector exhibits relatively better performance in accurately predicting radiation sources from the bottom left, bottom right, and top regions. Nevertheless, it is not as effective in handling radiation sources situated in the bottom center region.

These findings provide valuable insights into the strengths and limitations of each detector type, empowering researchers and practitioners to make informed decisions based on the specific application requirements and the expected location of radiation sources. Leveraging this knowledge, we can optimize the performance of radiation detection systems, enhancing their utility in various fields such as nuclear safety, medical imaging, and industrial inspections and ultimately contributing to developing safer and more efficient technologies for radiation detection.

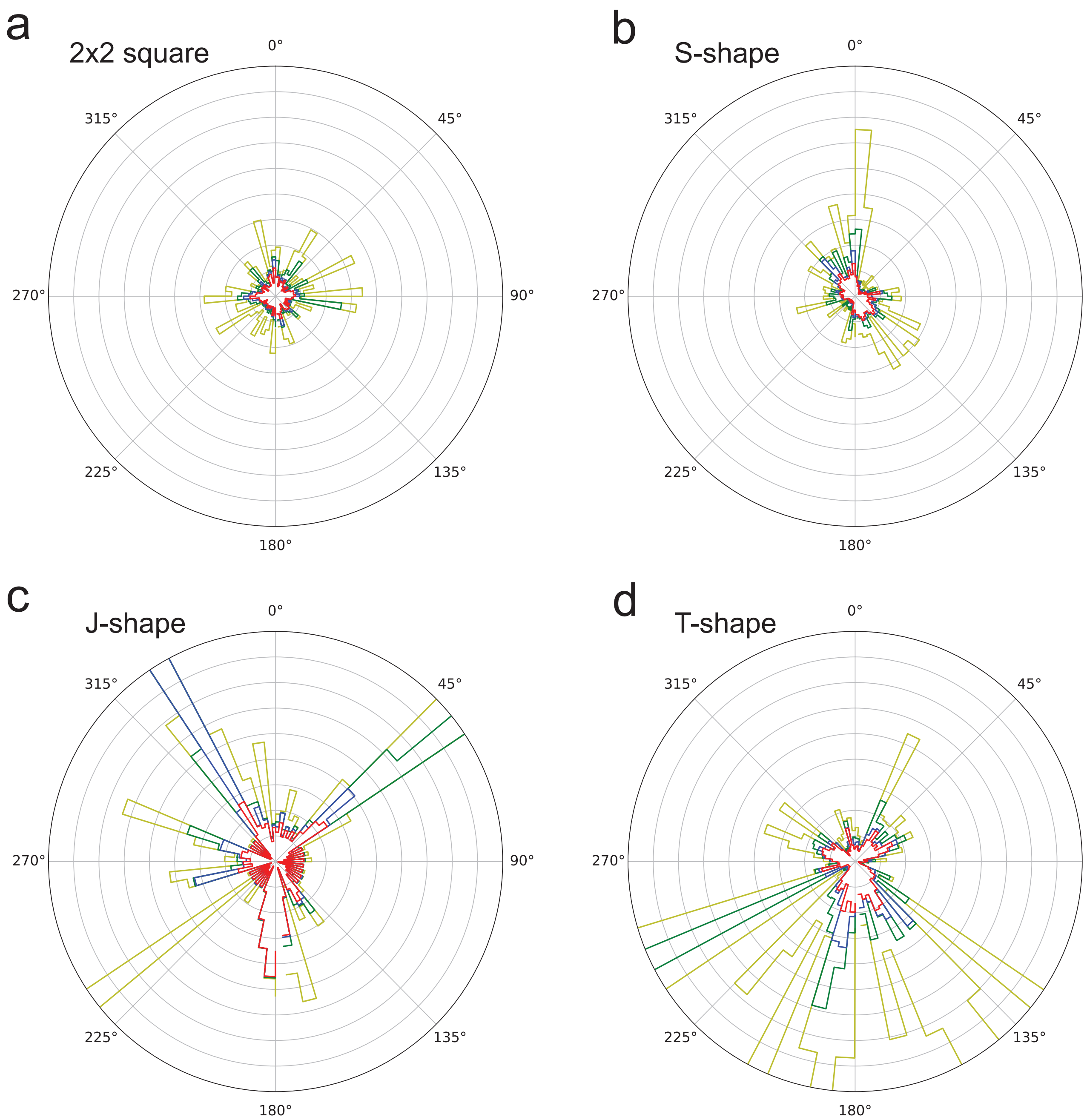

Figure S4. **Angular dependency of the directional predictions**
Polar axis plots of the Wasserstein distance loss for each using (a) 2×2 detector (b) S-shape (c) J-shape (d) T-shape detector. We show the errors predicting source direction when it is apart by 50 cm (red), 100 cm (blue), 200 cm (green), and 500 cm (yellow).

III.2 Gamma Energy Dependency

We conducted a comprehensive study to investigate the performance of radiation detectors under varying gamma radiation source energy values. We recognized that the energy of the radiation source could affect the prediction loss of the detectors. To explore this relationship, we trained our model with a specific source energy value of 0.5 MeV, serving as the baseline for our evaluations. Figure S5 presents a detailed analysis of the prediction errors by an S-shaped detector, obtained for source energies ranging from 0.1 MeV to 5 MeV, and plotted as a function of the source direction angle. This comprehensive visualization allowed us to observe how the prediction errors varied across different energy levels and angles, providing critical insights into the detectors' performances.

Our research revealed the trends regarding the influence of source energy on prediction loss. Below the source energy value of 0.5 MeV, we observed that the prediction error exhibited little to no dependence on the source energy. This finding is particularly encouraging, implying that the detectors can maintain consistent and accurate predictions for radiation sources with lower energy levels. However, as the source energy increased to 0.5 MeV or higher, we observed a distinct change in the prediction error behavior. The prediction error demonstrated an upward trend, getting larger as the energy (and therefore penetrability) of the photons increased. This outcome indicates that the detectors face greater challenges and complexities in accurately predicting the direction of radiation sources with higher energy levels. The insights gained from this study hold significant implications for the practical application of radiation detectors. Understanding the relationship between source energy and prediction loss is crucial for optimizing and calibrating detectors in real-world scenarios. By recognizing the energy dependence of the detectors' performance, we can make informed decisions when selecting and deploying radiation detection systems for specific applications.

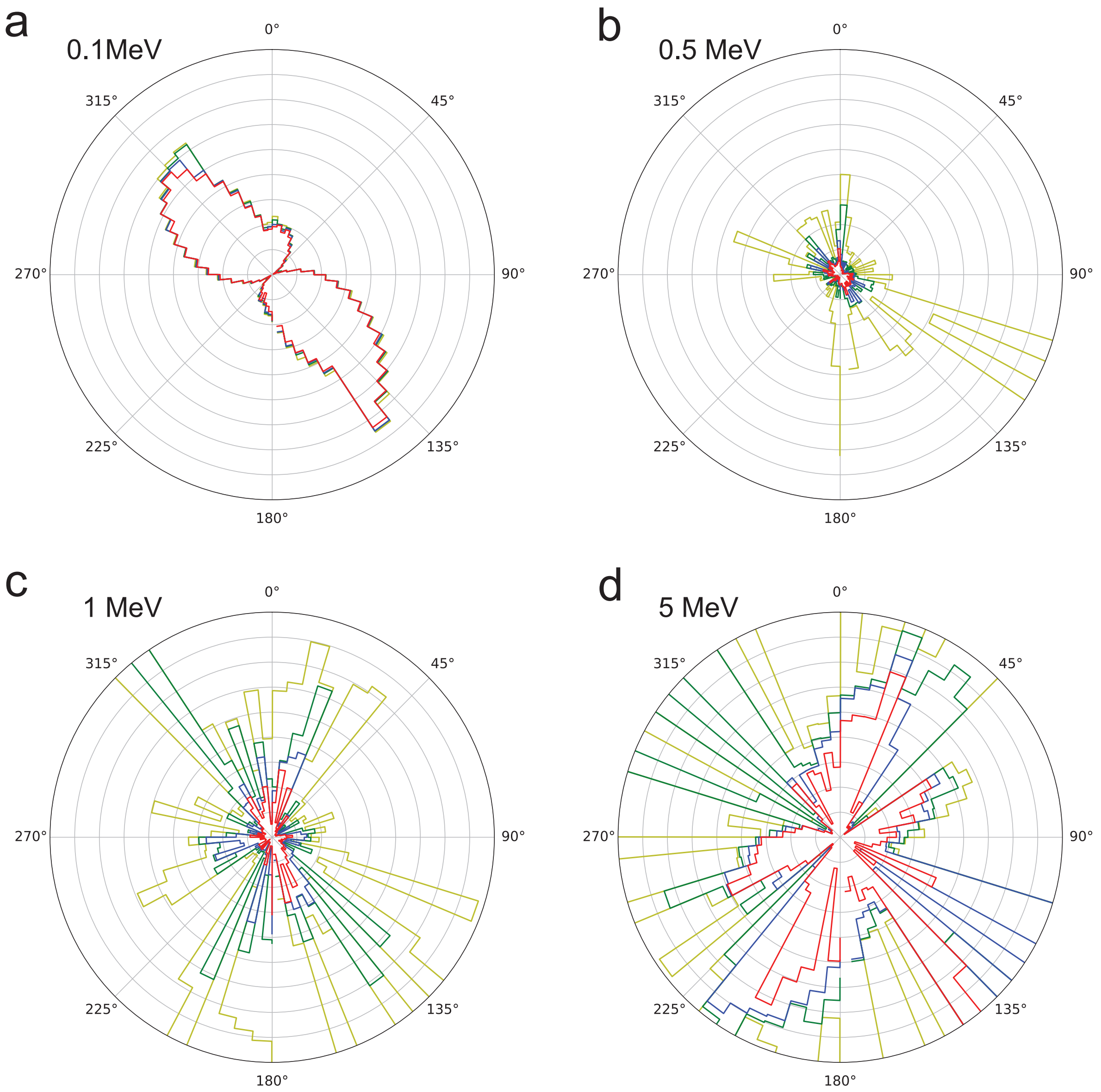

Figure S5. Energy dependency of the directional predictions
Polar axis plots of the Wasserstein distance loss for the cases using a radiation source of (a) 0.1 MeV (b) 0.5 MeV (c) 1 MeV (d) 5 MeV. We show the errors predicting source direction when it is apart by 50 cm (red), 100 cm (blue), 200 cm (green), and 500 cm (yellow). The S-shaped detector was used for the comparison with different radiation source energy values.

III.3 Directional Prediction of Two Radiation Sources with Single Filter Layer Model

The potential of our Tetris-inspired detector in localizing radiation sources is promising. Nonetheless, the task of predicting the directions becomes increasingly intricate with the escalation in the number of radiation sources due to the constraints presented by the number of input signals.

In addressing this challenge, we surveyed deep into the detector's capability to locate multiple radiation sources. The efficacy of detection hinges significantly on the detector's configuration and the count of detector panels employed. We introduced specific conditions for directional prediction to manage the complexities. The distance between the radiation source and the detector was consistently maintained at 200 cm, with only the directions of the radiation sources varying. Essentially, we visualized a circle with a 200 cm radius encircling the detector where two radiation sources were positioned. The training paradigm was designed to enable the model to pinpoint only the direction of these two radiation sources, not to predict the distances from the detector to the radiation sources. By stabilizing the source-detector distance, we eliminated the need for dual filter layers. Subsequently, we adopted a singular filter layer and reduced the number of input channels to one. This operating principle emanates from our postulation that even with the most basic configuration of four detector panels, it's plausible to discern the directions of two radiation sources. In other word, here we aim to solve the two variables given 4 detector readings. We executed 4,000 simulations for each detector by modulating the source directions. The data set was apportioned into training (90%) and testing (10%) datasets.

Figure S6 elucidates the comparative results of direction predictions for two radiation sources employing the static detector. The scenarios depicted with detectors consisting of 4 panels are portrayed for each row: 2×2 square detector (Fig.S6a-c), S-shape (Fig.S6d-f), J-shape (Fig.S6g-i), and T-shape (Fig.S6j-l). Table S3 aggregates the data, showcasing the largest, smallest, and average 1-Wasserstein distance. Although the prediction error can get large in some cases, we could find that even detectors equipped with a mere four panels manifest the capability to capture directional information. This assertion is corroborated by Figure S6, where the peak positions of the predicted angles align closely with the ground truth. In summation, our findings underscore that even detectors with simple configurations can adeptly learn the characteristics of radiation source directions, given the readouts of the detectors.

Table S3. Ablation on neural network backbones with single filter layer. We predicted the directions of two radiation sources placed at 200 cm from the detector. We demonstrated different scenarios (2×2 square grid and three types of Tetrominos), evaluated by the Wasserstein distance of angular distributions.The table shows the lowest, highest, and average prediction losses, and the angles show the errors in the bracket, respectively. Here, we consider the case the distance between the detector and the radiation source is 200 cm.

	2×2	S-shape	J-shape	T-shape
Largest error	14.31103	14.99088	16.51298	13.60041
Smallest error	0.99164	0.84007	0.89351	0.68228
Average error	4.59137	4.93340	4.99131	4.17611

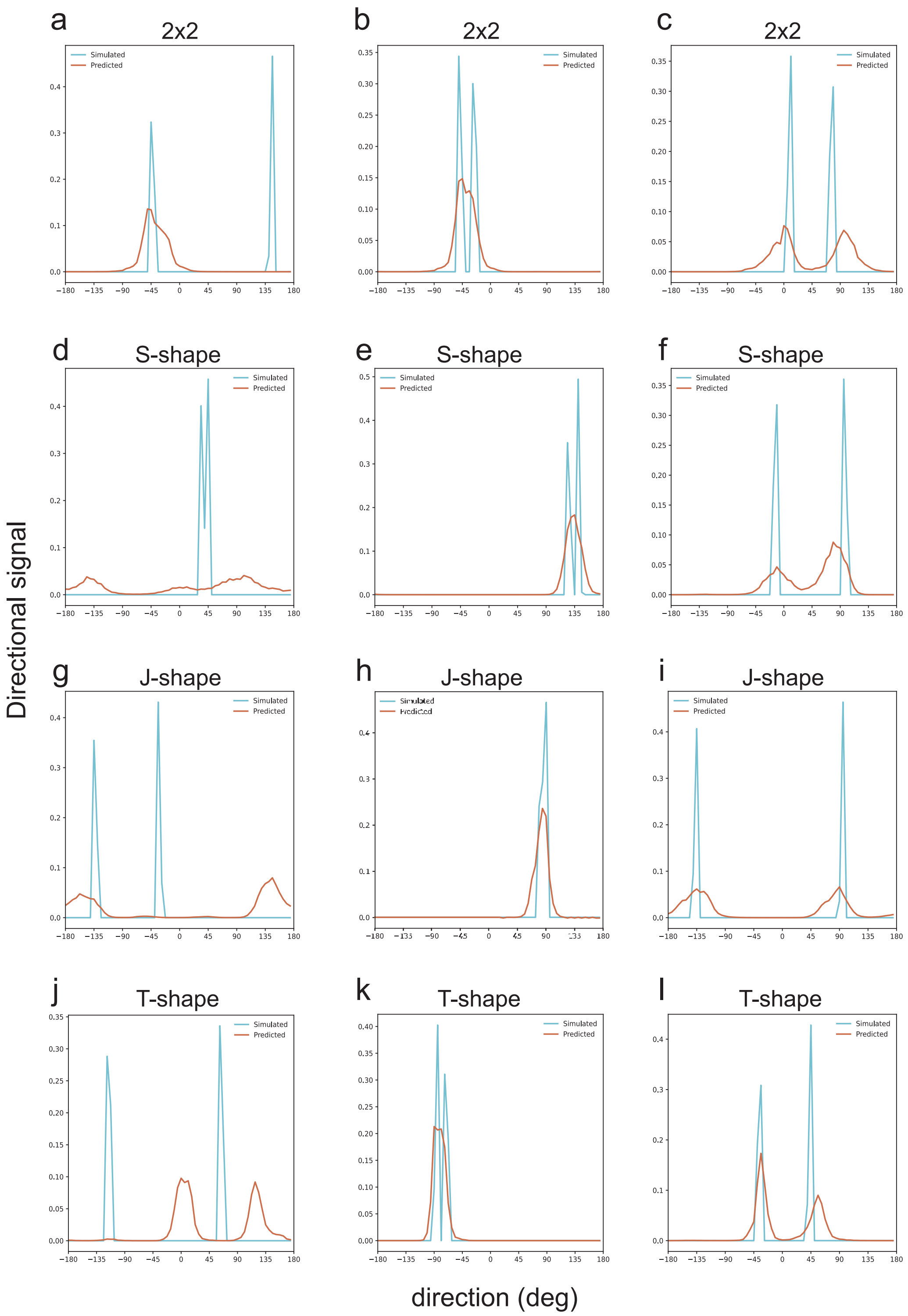

Figure S6. Data of directional prediction for two radiation Sources using different detector geometries
The figure presents the results of directional prediction experiments involving two radiation sources, each observed using various detector geometries. The left column (**a, d, g, j**) depicts the directional prediction outcomes with the largest prediction loss among the sources, showcasing detectors of square 2×2 shape, S-shape, J-shape, and T-shape, respectively. In the middle column (**b, e, h, k**), the directional prediction results with the smallest prediction loss are illustrated for the same detector geometries. Notably, the right column (**c, f, i, l**) showcases instances where detectors accurately capture the directions of two separated radiation sources. The detector geometries include square 2×2 detectors (**a-c**), S-shape detectors (**d-f**), J-shape detectors (**g-i**), and T-shape detectors (**j-l**). These results provide insights into the varying performance of detectors under different scenarios, shedding light on their predictive capabilities and limitations.

It is meaningful to conduct the comparative study seeking to understand how different filter layer configurations impact the accuracy of predicting radiation source directions. To examine the role of filter layers, we designed and trained three distinct models, each offering a different perspective on capturing source direction information: (i) Two-Filter Layer Model: This model harnesses both near-field (distance: 50 cm) and far-field (distance: 500 cm) filters. We aim to capture nuances in radiation direction prediction across various distances by incorporating both filters. (ii) Single Near-Field Filter Model: This model focuses solely on the near-field filter, aiming to excel in capturing directional cues from proximity to the detector. (iii) Single Far-Field Filter Model: Conversely, this model exclusively relies on the far-field filter to specialize in predicting radiation direction when the source is comparatively distant.

To gauge the performance of these models, we conducted extensive training and testing using the common dataset. The outcomes of this comparative analysis are summarized in Table S4, showcasing the average prediction error for each model. Intriguingly, a distinct pattern emerged from the results: Models utilizing a single filter layer, particularly the far-field filter variant, exhibited notably lower prediction errors than the two-filter layer model.

Table S4. The average directional prediction error using static detector (2×2 square) using two or one filter layers.

	Two-Filter Layer Model	Single Near-Field Filter Model	Single Far-Field Filter Model
Average test error	1.08844	0.88746	0.87561

Our investigation into the role of filter layers in radiation detection models sheds light on the complex interplay between architecture and accuracy. While single-filter layer models excel in specialized prediction, the two-filter layer model offers capability in accommodating diverse scenarios. Given the primary objective of predicting radiation source directions, eliminating components tied to source distance seems to enhance the specialized prediction of source directions. This suggests that such models concentrate on the most essential directional features while disregarding factors irrelevant to the primary goal. On the other hand, the two-filter layer model offers its own merits. The proposed approach of integrating multiple filter layers proves advantageous in effectively segregating features linked to radiation sources at varying distances. This feature separation contributes to enhanced accuracy as the model learns to discriminate and employ features depending on the positional relations of radiation sources.

III.4 Effect of Background Noise

Detecting radiation sources accurately requires careful consideration of background noise. The inherent variability in the signals captured by individual detector pixels introduces uncertainties in the directional prediction results. To understand the implications of background noise, we embarked on an empirical investigation aimed at unraveling the connection between noise levels and prediction accuracy.

Following the central limit theorem, which suggests that the distribution of noise can become approximately Gaussian regardless of the original distribution, we postulate that a Gaussian distribution can approximate the Poisson-distributed background noise, assuming high enough counts of input photon. Our investigation employed signals obtained through OpenMC simulations from each pixel of the detector panels. These signals were then normalized to adhere to a normal distribution. We systematically introduced Gaussian noise with varying levels of variance σ^2 to the normalized detector input signals. The resulting noisy data served as our test dataset, and the prediction models were trained on noise-free training data.

Figure S7 presents a comprehensive overview of the relationship between noise levels and prediction errors across different detectors. As the noise level escalates, prediction errors tend to increase consistently. This observation emphasizes the adverse impact of background noise on the accuracy of radiation source prediction. Higher noise levels introduce uncertainty into the detected signal, thereby hampering the precision of the directional predictions. It is intriguing to observe variations in prediction behavior among different detector shapes in the presence of background noise. The S-shape detector, for instance, outperforms the square detector when subjected to minimal or negligible noise levels. This performance difference underscores the capability of the S-shape detector to capture subtle variations even in the presence of low-level noise. However, Tetris-inspired detectors (S-, J-, T- shapes) exhibit a more pronounced escalation in prediction error as the noise distribution widens. This differential behavior underscores that while some detectors might fare better under certain noise conditions, Tetris-inspired detectors are particularly susceptible to the deleterious effects of background noise.

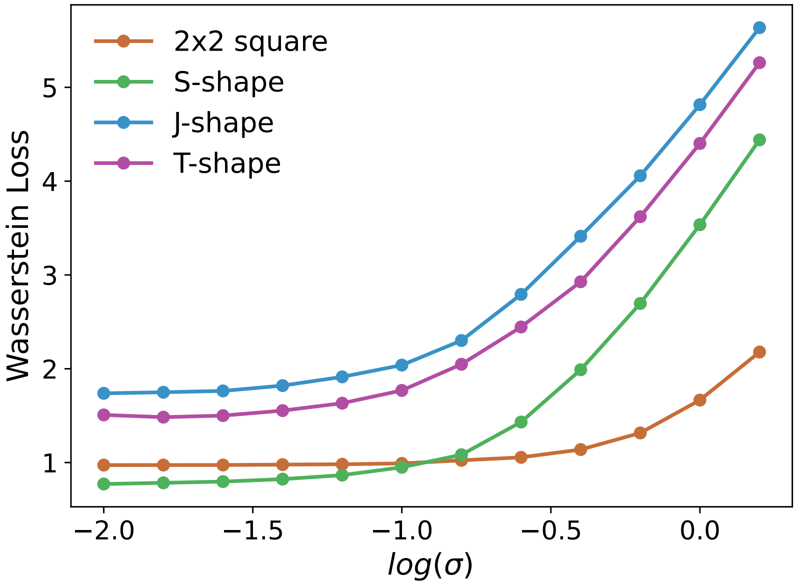

Figure S7. **Relationship between background noise and prediction errors for different detectors.**
Random noise with variance σ^2 are added to the test data of 4-panel detectors. The average prediction error is plotted for each $\log(\sigma)$ value.

IV Supplementary Note 4: Radiation Source Mapping with Maximum A Posteriori (MAP) Estimation

A method based on Maximum a Posteriori (MAP) Estimation is applied to generate the radiation distribution map. In the mapping process, the intensity contribution of the ith pixel to the jth directional sector of the detector at time t is recorded. Figure S8 explains the roles of these factors in the mapping process. Using this method, we have worked on single radiation source detection using detectors with four CZT panels. The result using the S-shape detector, which worked the best among the other simple configurations, is shown in our main document. Figure S9-S11 illustrate the radiation mapping results with 2×2 square, J- and T-shape detectors. To track the directional prediction at each time during the mapping process, we plot the direct predictions of angles in Fig. S12. It is obvious that the fluctuation of the directional predictions at each site reflects the performance of radiation mapping. Furthermore, we have worked on mapping two radiation sources simultaneously. Figure S13-S14 show the result using 10×10 and 5×5 square detector.

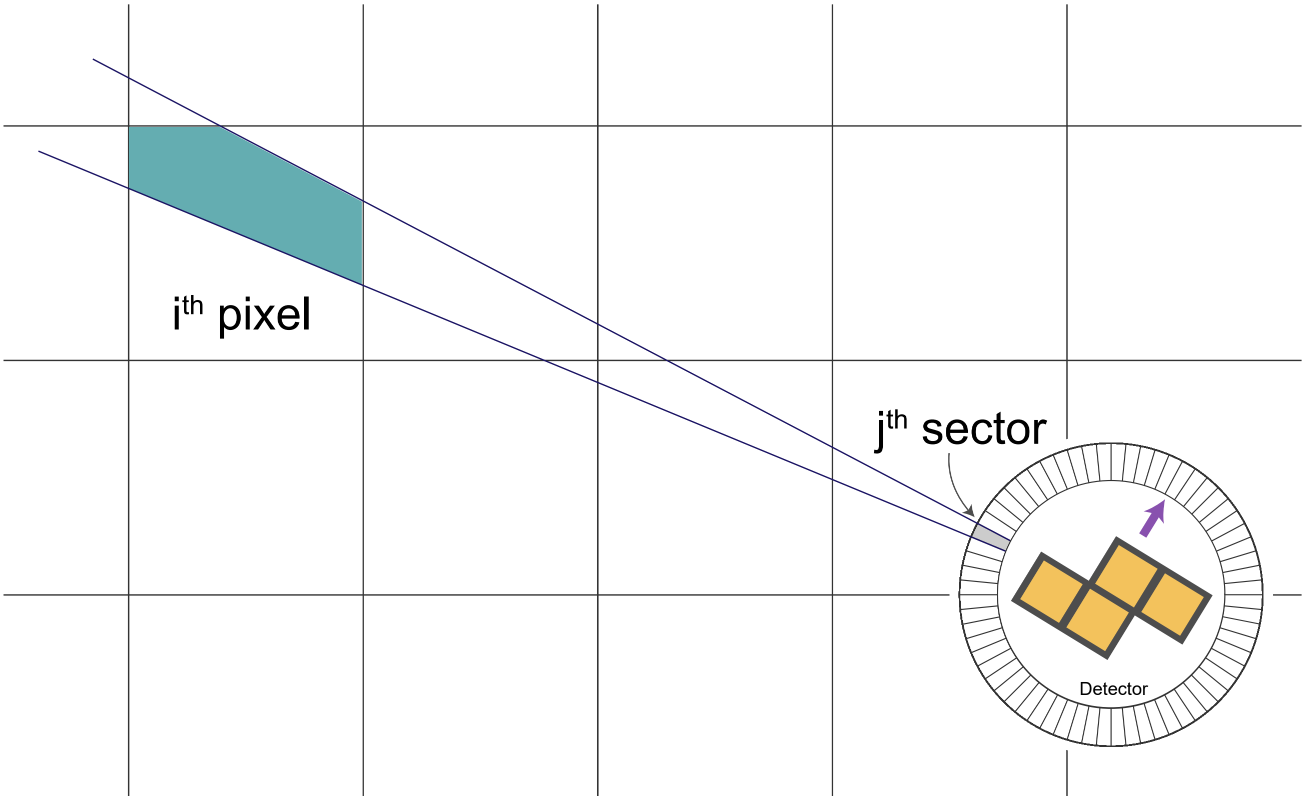

Figure S8. An illustration of the element of the observation matrix.
The purple arrow near the detector indicates the front side of the detector. The blue area on the grid indicates the overlapped region of the ith pixel and the jth sector of the detection angle.

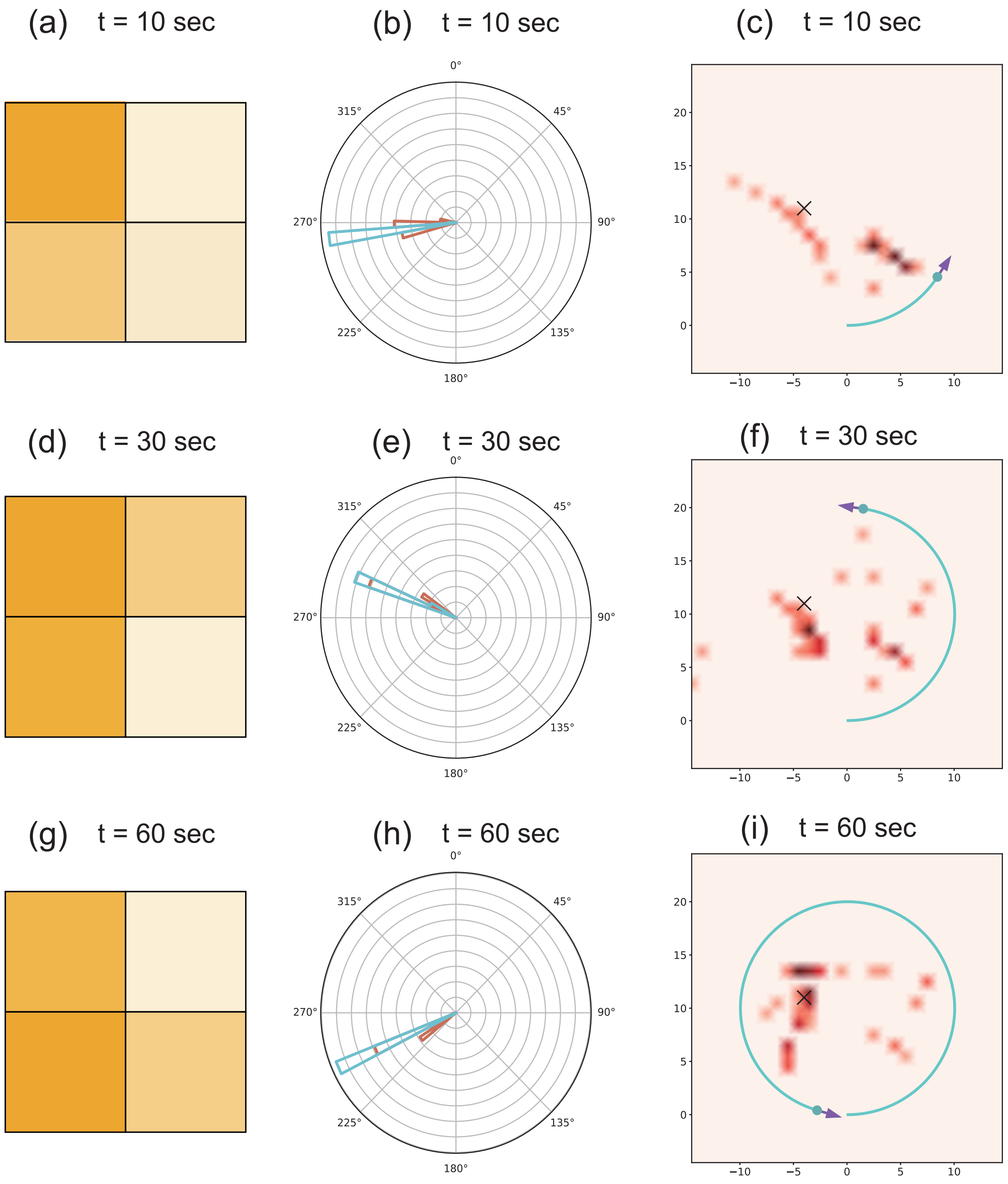

Figure S9. Radiation mapping with a 2×2 square detector.
a,d,g. The detector's input signals at t=10, 30, 60 sec. The panels with intense colors represent larger values on the detector's signal. The top side of the detector represents the front side of the moving detector. **b,e,h.** The predicted direction of the radiation source at t=10, 30, 60 sec. On the polar coordinates, the blue and brown curves represent the ground-truth and predicted direction of the radiation source. **c,f,i.** The process to map the radiation source at t=10, 30, 60 sec. The "×" symbols on the maps show the correct position of the radiation source. The purple arrows indicate the front side of the moving detector. Areas with intense red colors indicate sites with a high probability of possessing radiation sources. The unit of length is the meter. Check the radiation mapping process in Supplementary Movie 2.

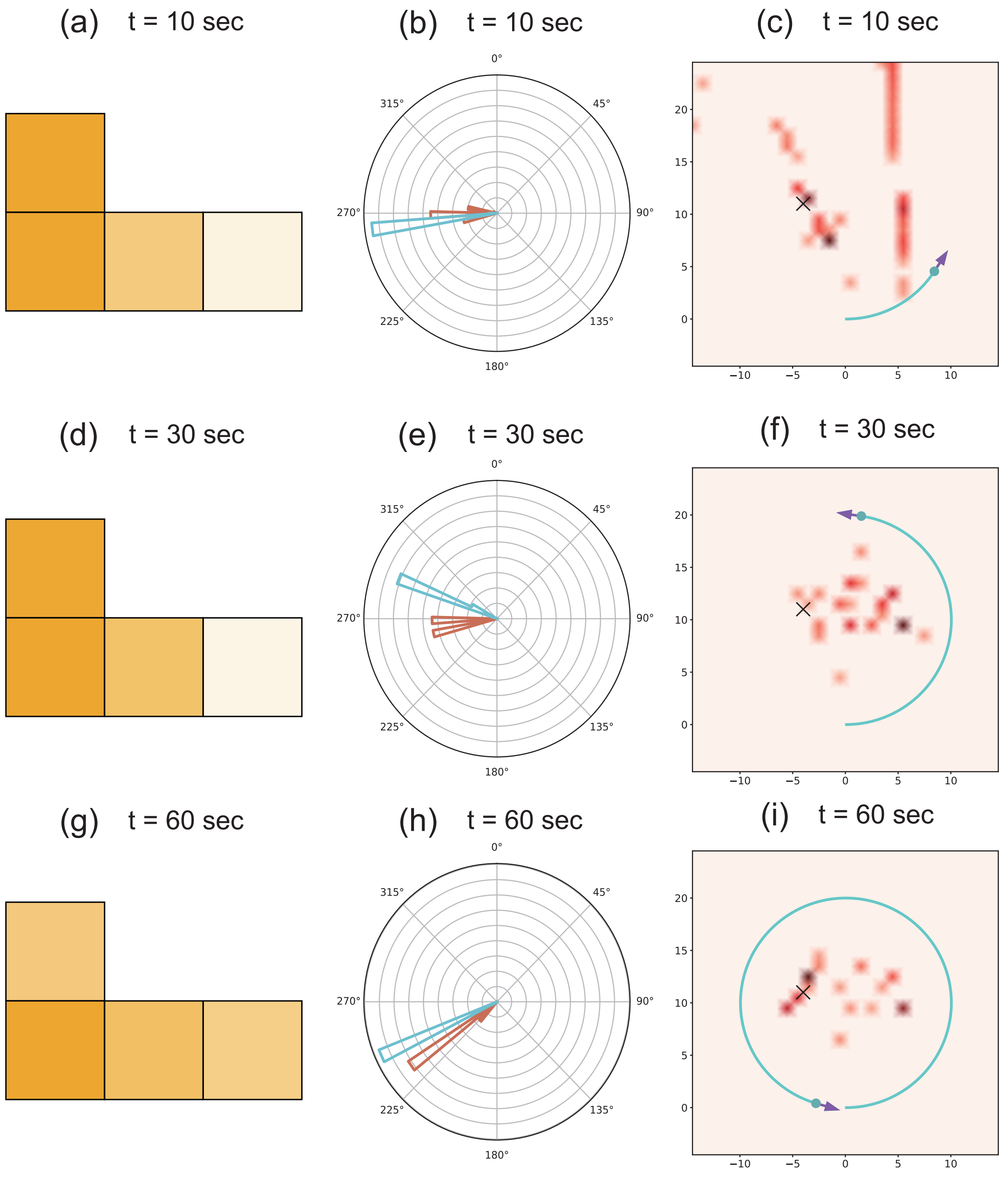

Figure S10. **Radiation mapping with a J-shape Tetris-inspired detector.**
a,d,g. The detector's input signals at t=10, 30, 60 sec. The panels with intense colors represent larger values on the detector's signal. The top side of the detector represents the front side of the moving detector. **b,e,h.** The predicted direction of the radiation source at t=10, 30, 60 sec. On the polar coordinates, the blue and brown curves represent the ground-truth and predicted direction of the radiation source. **c,f,i.** The process to map the radiation source at t=10, 30, 60 sec. The "×" symbols on the maps show the correct position of the radiation source. The purple arrows indicate the front side of the moving detector. Areas with intense red colors indicate sites with a high probability of possessing radiation sources. The unit of length is the meter. Check the radiation mapping process in Supplementary Movie 3.

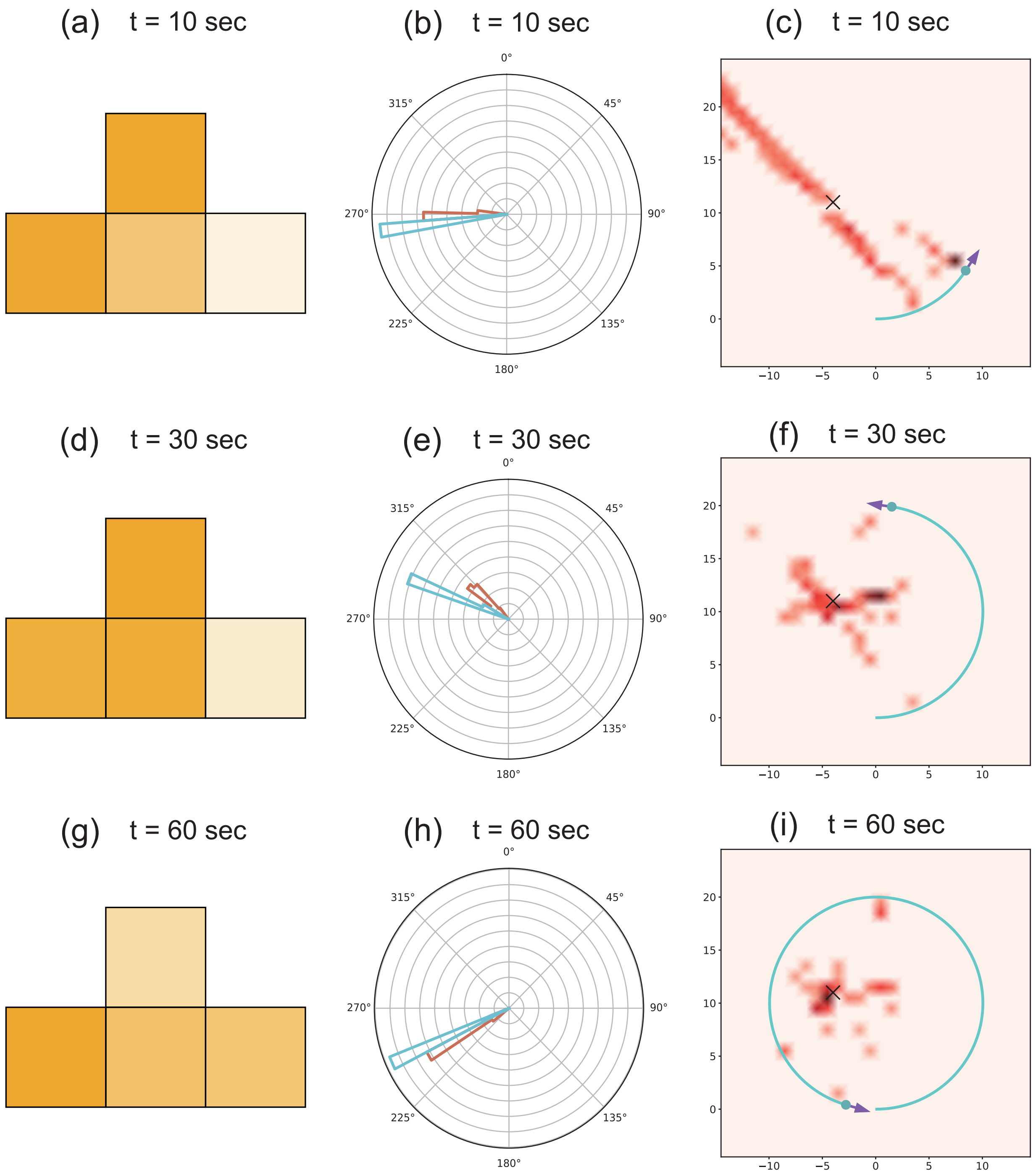

Figure S11. **Radiation mapping with a T-shape Tetris-inspired detector.**
a,d,g. The detector's input signals at t=10, 30, 60 sec. The panels with intense colors represent larger values on the detector's signal. The top side of the detector represents the front side of the moving detector. **b,e,h.** The predicted direction of the radiation source at t=10, 30, 60 sec. On the polar coordinates, the blue and brown curves represent the ground-truth and predicted direction of the radiation source. **c,f,i.** The process to map the radiation source at t=10, 30, 60 sec. The "×" symbols on the maps show the correct position of the radiation source. The purple arrows indicate the front side of the moving detector. Areas with intense red colors indicate sites with a high probability of possessing radiation sources. The unit of length is the meter. Check the radiation mapping process in Supplementary Movie 4.

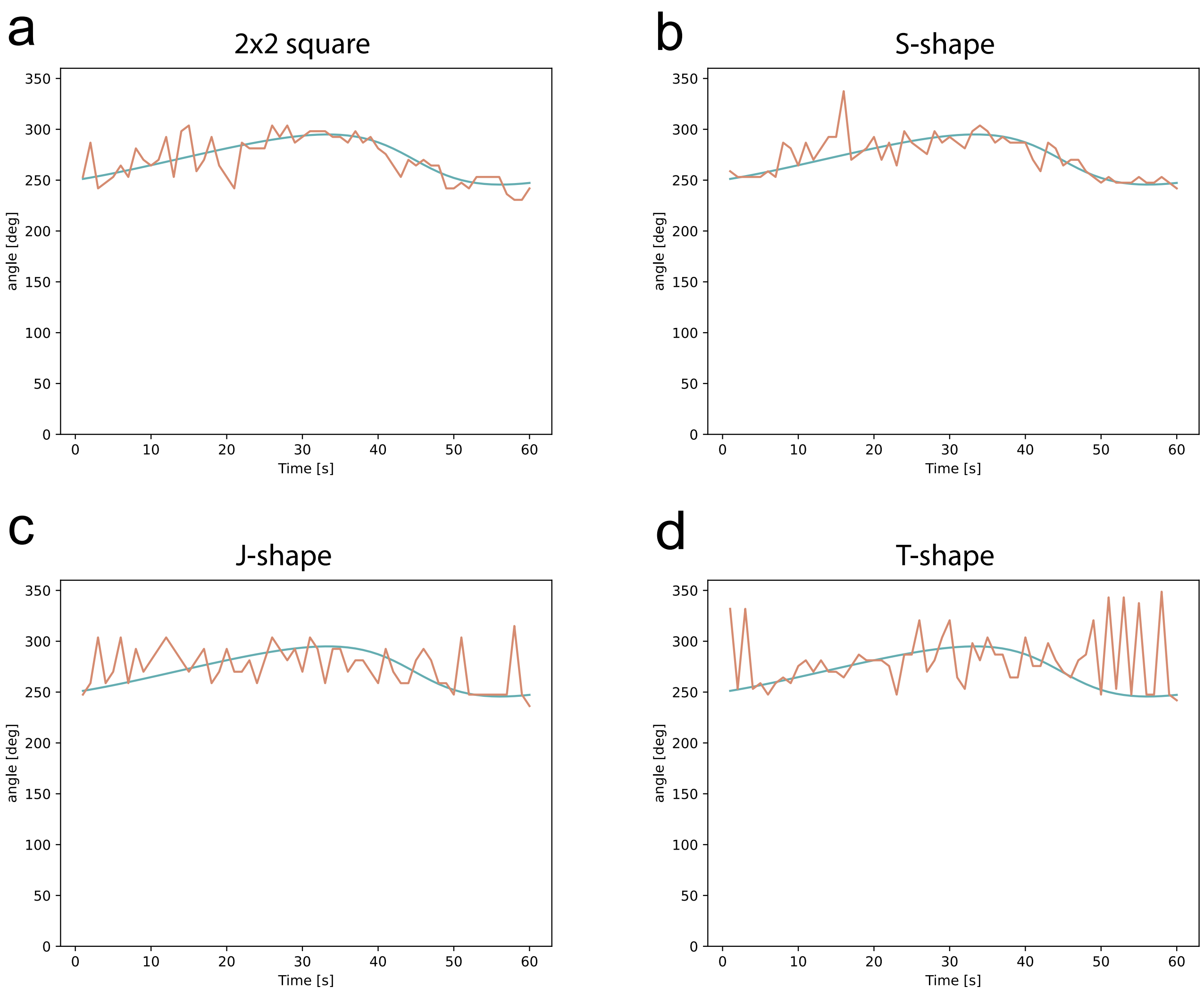

Figure S12. The predicted and actual directions of the radiation source position.
The blue and brown lines track the changes of the actual directions and the predicted directions with detectors of **a.** 2×2 square shape **b.** S-shape **c.** J-shape **d.** T-shape during the radiation mapping process of Figure 3, S9-S11. Here, the direction is defined as the clockwise angle from the front side of the detector.

V Supplementary Note 5: Mapping Two Radiation Sources

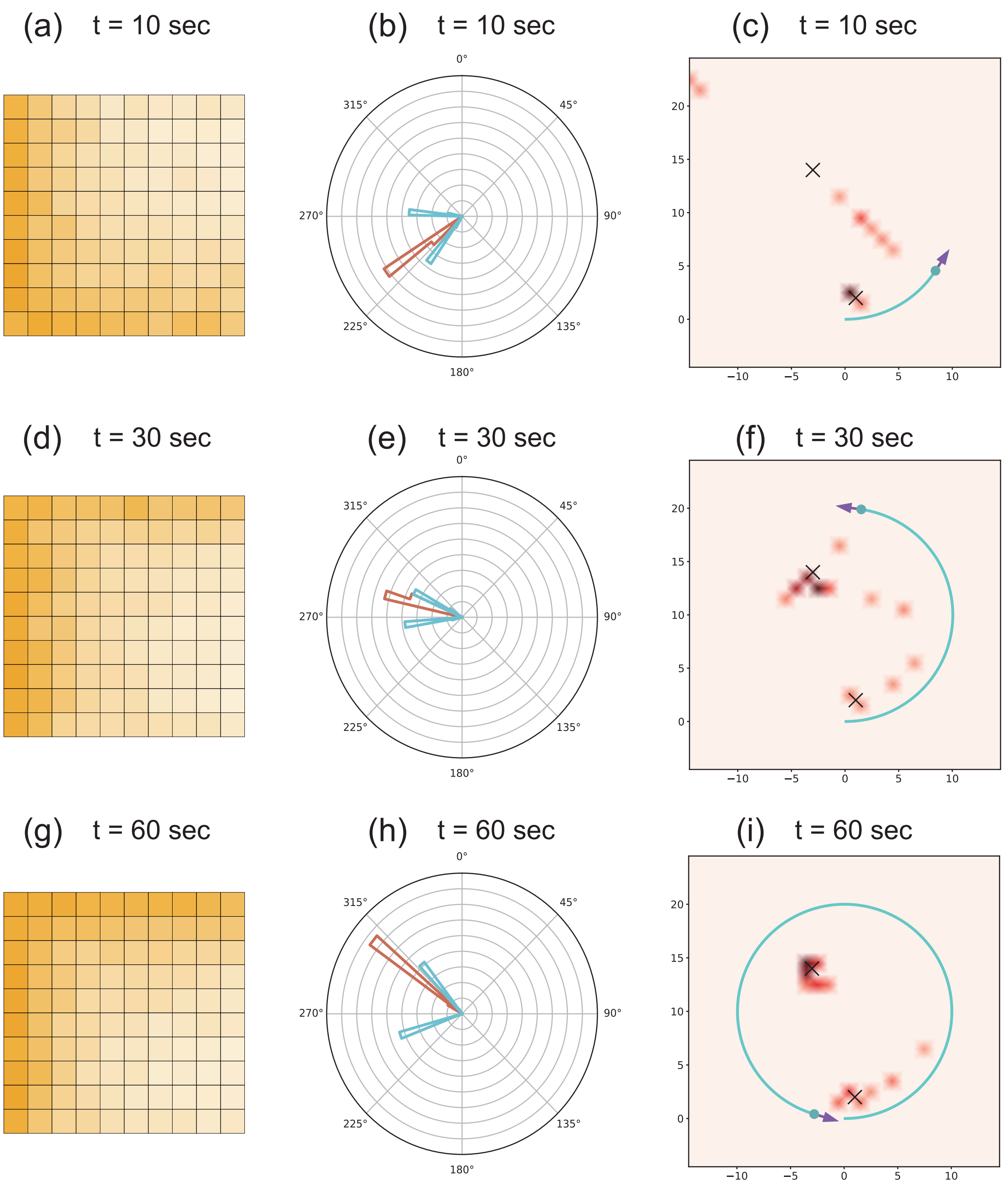

Figure S13. Mapping 2 radiation sources with a 10×10-size detector.
a,d,g. The detector's input signals at t=10, 30, 60 sec. The panels with intense colors represent larger values on the detector's signal. The top side of the detector represents the front side of the moving detector. **b,e,h.** The predicted direction of the radiation source at t=10, 30, 60 sec. On the polar coordinates, the blue and brown curves represent the ground-truth and predicted direction of the radiation source. **c,f,i.** The process to map the radiation source at t=10, 30, 60 sec. The "×" symbols on the maps show the correct position of the radiation source. The purple arrows indicate the front side of the moving detector. The area with intense red colors indicates the sites with a high probability of possessing radiation sources. The unit of length is the meter. Check the radiation mapping process in Supplementary Movie 5.

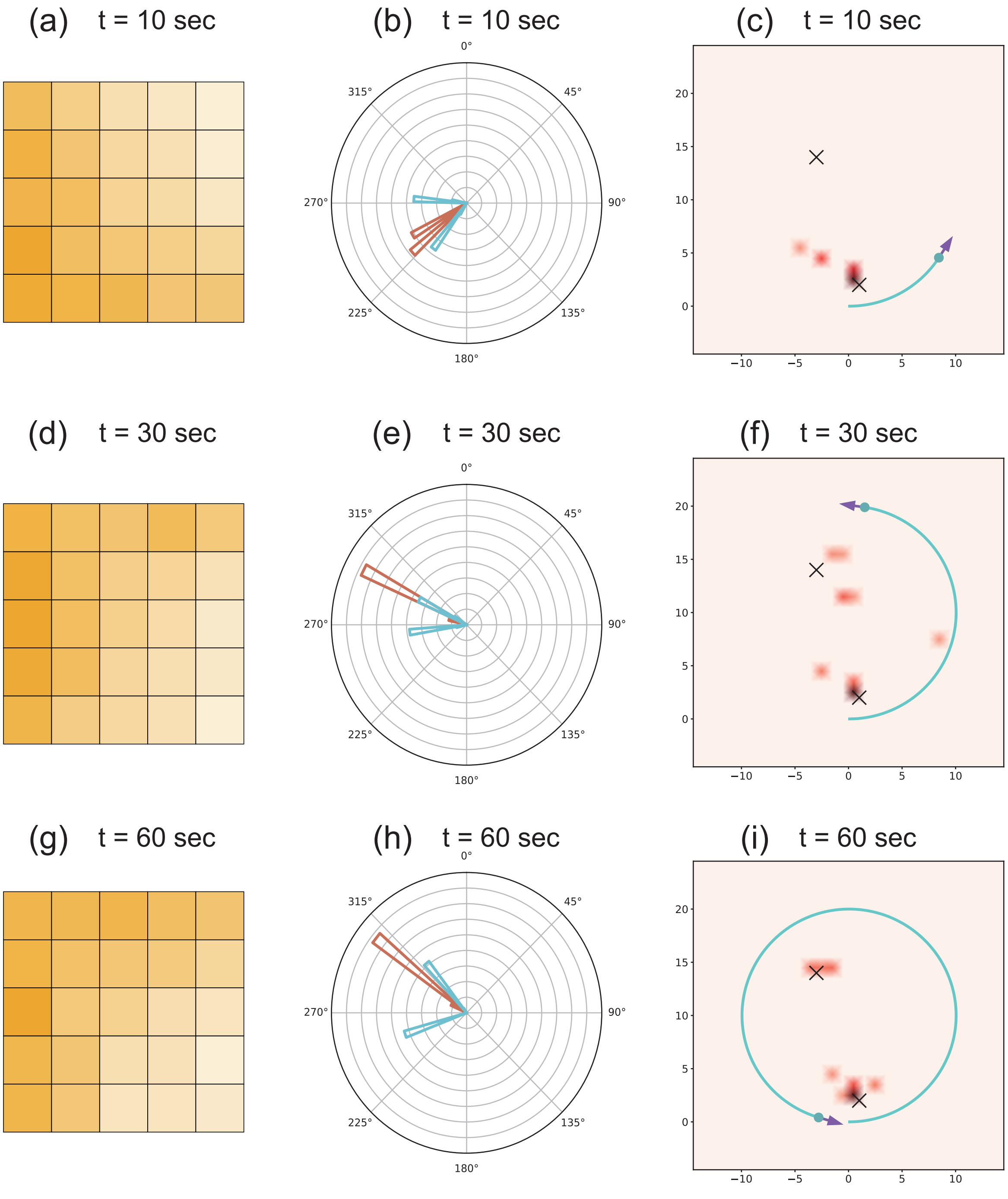

Figure S14. Mapping 2 radiation sources with a 5×5-size detector.
a,d,g. The detector's input signals at t=10, 30, 60 sec. The panels with intense colors represent larger values on the detector's signal. The top side of the detector represents the front side of the moving detector. **b,e,h.** The predicted direction of the radiation source at t=10, 30, 60 sec. On the polar coordinates, the blue and brown curves represent the ground-truth and predicted direction of the radiation source. **c,f,i.** The process to map the radiation source at t=10, 30, 60 sec. The "×" symbols on the maps show the correct position of the radiation source. The purple arrows indicate the front side of the moving detector. Areas with intense red colors indicate sites with a high probability of possessing radiation sources. The unit of length is the meter. Check the radiation mapping process in Supplementary Movie 6.

VI Supplementary Note 6: Radiation Mapping with a Rotating Detector Moving along the Circular Trajectory.

In the previous section we have shown that the detectors which are always facing the traveling direction can map radiation sources. However, it is also worthwhile mentioning that the particular detector face that aligns with the detector's moving direction does not matter much. The detector facing any direction is already a valid directional detector sensitive to radiations coming from all directions. We demonstrate the cases in which the moving S-shape detector does not face the traveling direction but rotates at its sites along the circular trajectory. Figure S15 shows how we set up the simulation. As the detector moves along the circular trajectory by angle θ counterclockwise, we rotate the detector by φ counterclockwise. Figure S16-S19 show the mapping results with $\varphi = \theta, 2\theta, -\theta, -2\theta$. Note that $\varphi = \theta$ means the facing side of the detector looks constant from the static observer. We plot the direct predictions of angles in Fig. S20, as we did in the previous section.

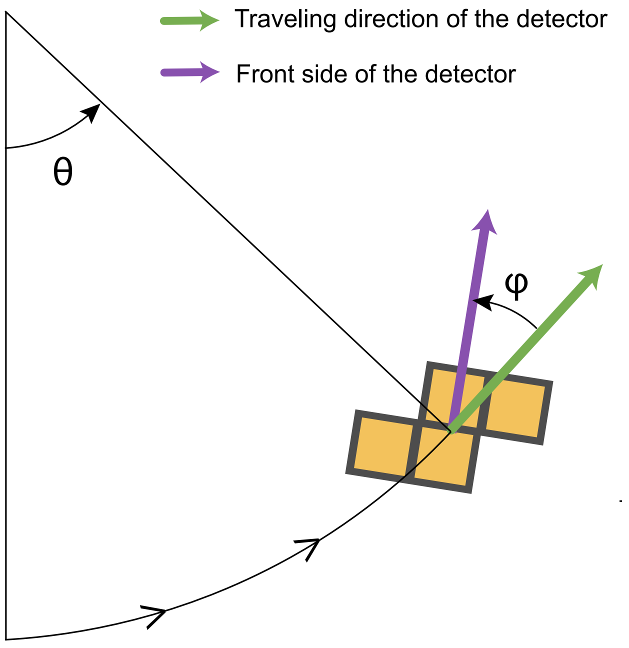

Figure S15. The method to rotate the directional detector when mapping the radiation source distributions. As the detector moves along the circular trajectory by angle θ counterclockwise, we rotate the detector by φ counterclockwise. The purple and green arrows indicate the front side of the moving detector and the traveling direction, respectively.

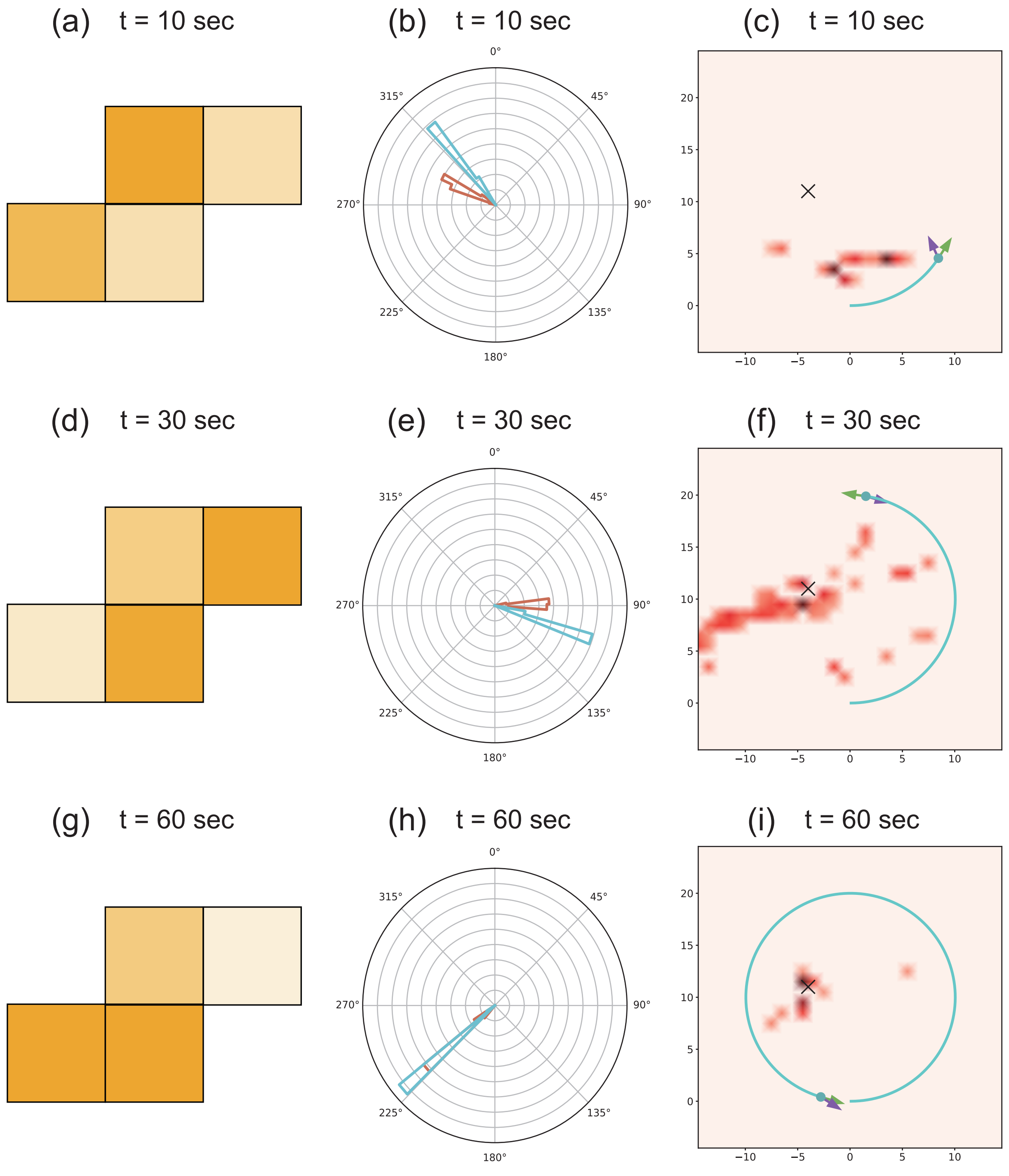

Figure S16. Radiation mapping with an S-shape Tetris-inspired detector ($\varphi = \theta$).
a,d,g. The detector's input signals at t=10, 30, 60 sec. The panels with intense colors represent larger values on the detector's signal. The top side of the detector represents the front side of the moving detector. **b,e,h.** The predicted direction of the radiation source at t=10, 30, 60 sec. On the polar coordinates, the blue and brown curves represent the ground-truth and predicted direction of the radiation source. **c,f,i.** The process to map the radiation source at t=10, 30, 60 sec. The "×" symbols on the maps show the correct position of the radiation source. The purple and green arrows indicate the front side of the moving detector and the traveling direction, respectively. Areas with intense red colors indicate sites with a high probability of possessing radiation sources. The unit of length is the meter. Check the radiation mapping process in Supplementary Movie 7.

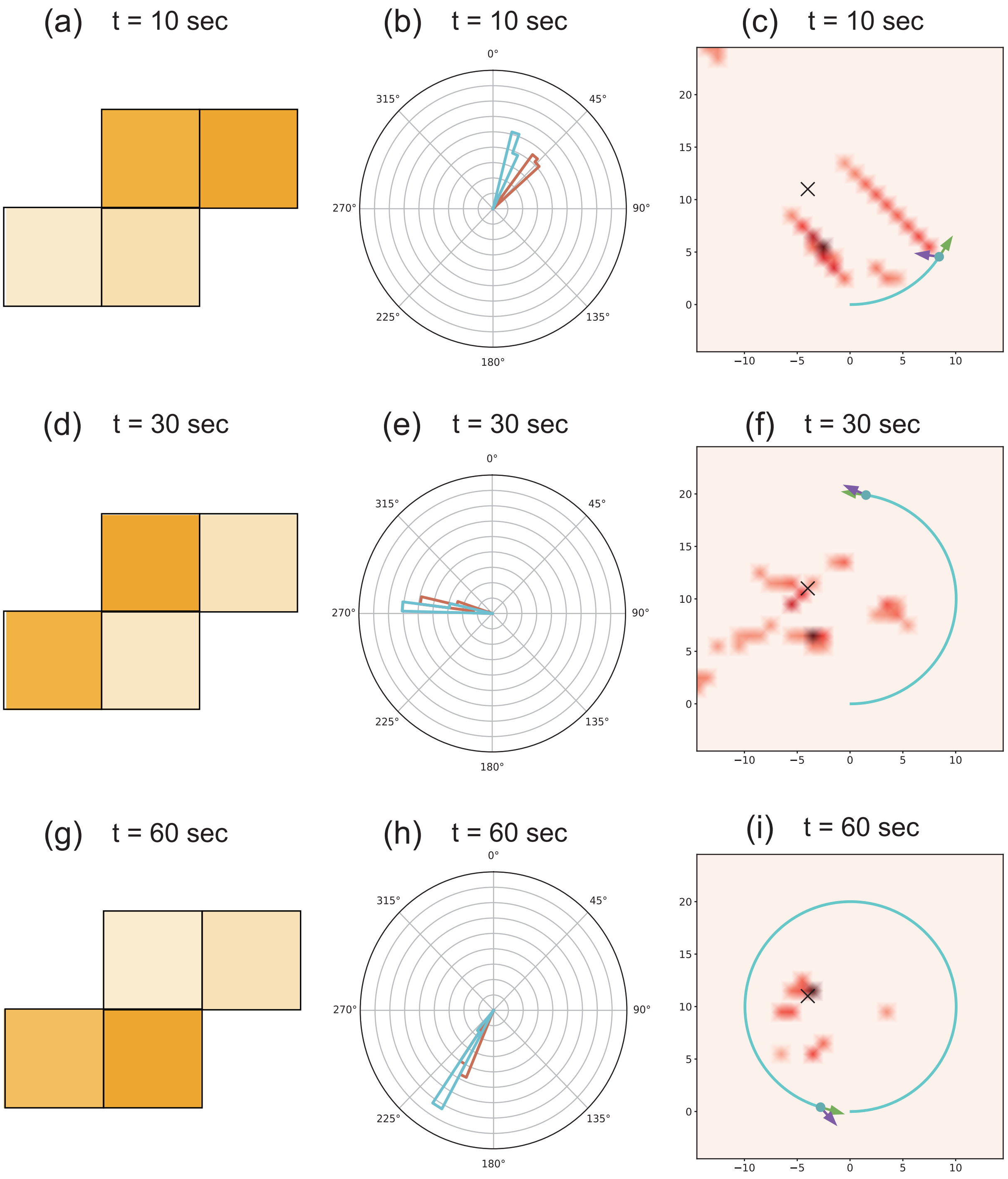

Figure S17. Radiation mapping with an S-shape Tetris-inspired detector ($\varphi = 2\theta$).
a,d,g. The detector's input signals at t=10, 30, 60 sec. The panels with intense colors represent larger values on the detector's signal. The top side of the detector represents the front side of the moving detector. **b,e,h.** The predicted direction of the radiation source at t=10, 30, 60 sec. On the polar coordinates, the blue and brown curves represent the ground-truth and predicted direction of the radiation source. **c,f,i.** The process to map the radiation source at t=10, 30, 60 sec. The "×" symbols on the maps show the correct position of the radiation source. The purple and green arrows indicate the front side of the moving detector and the traveling direction, respectively. Areas with intense red colors indicate sites with a high probability of possessing radiation sources. The unit of length is the meter. Check the radiation mapping process in Supplementary Movie 8.

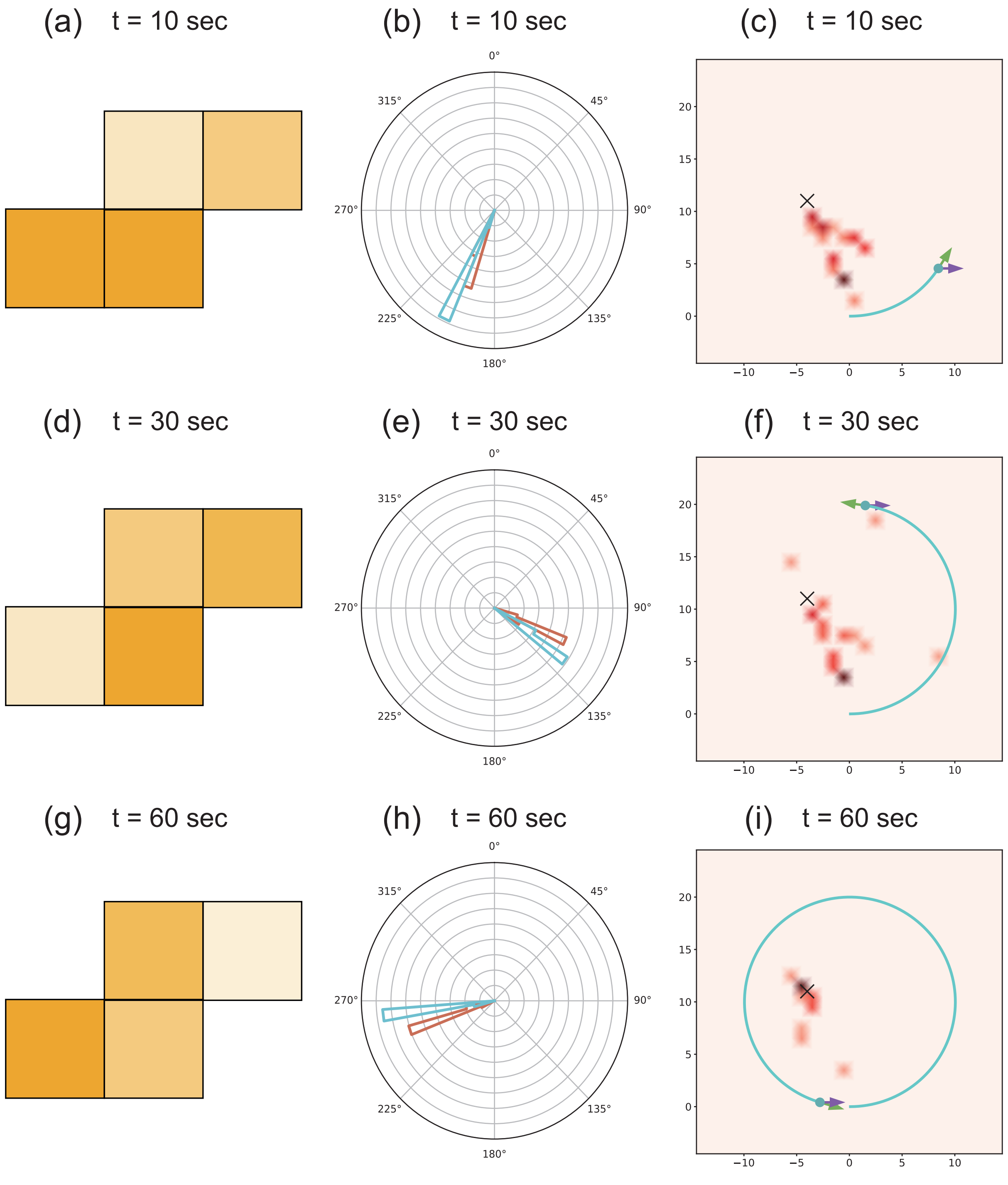

Figure S18. Radiation mapping with an S-shape Tetris-inspired detector ($\varphi = -\theta$).
a,d,g. The detector's input signals at t=10, 30, 60 sec. The panels with intense colors represent larger values on the detector's signal. The top side of the detector represents the front side of the moving detector. **b,e,h.** The predicted direction of the radiation source at t=10, 30, 60 sec. On the polar coordinates, the blue and brown curves represent the ground-truth and predicted direction of the radiation source. **c,f,i.** The process to map the radiation source at t=10, 30, 60 sec. The "×" symbols on the maps show the correct position of the radiation source. The purple and green arrows indicate the front side of the moving detector and the traveling direction, respectively. Areas with intense red colors indicate sites with a high probability of possessing radiation sources. The unit of length is the meter. Check the radiation mapping process in Supplementary Movie 9.

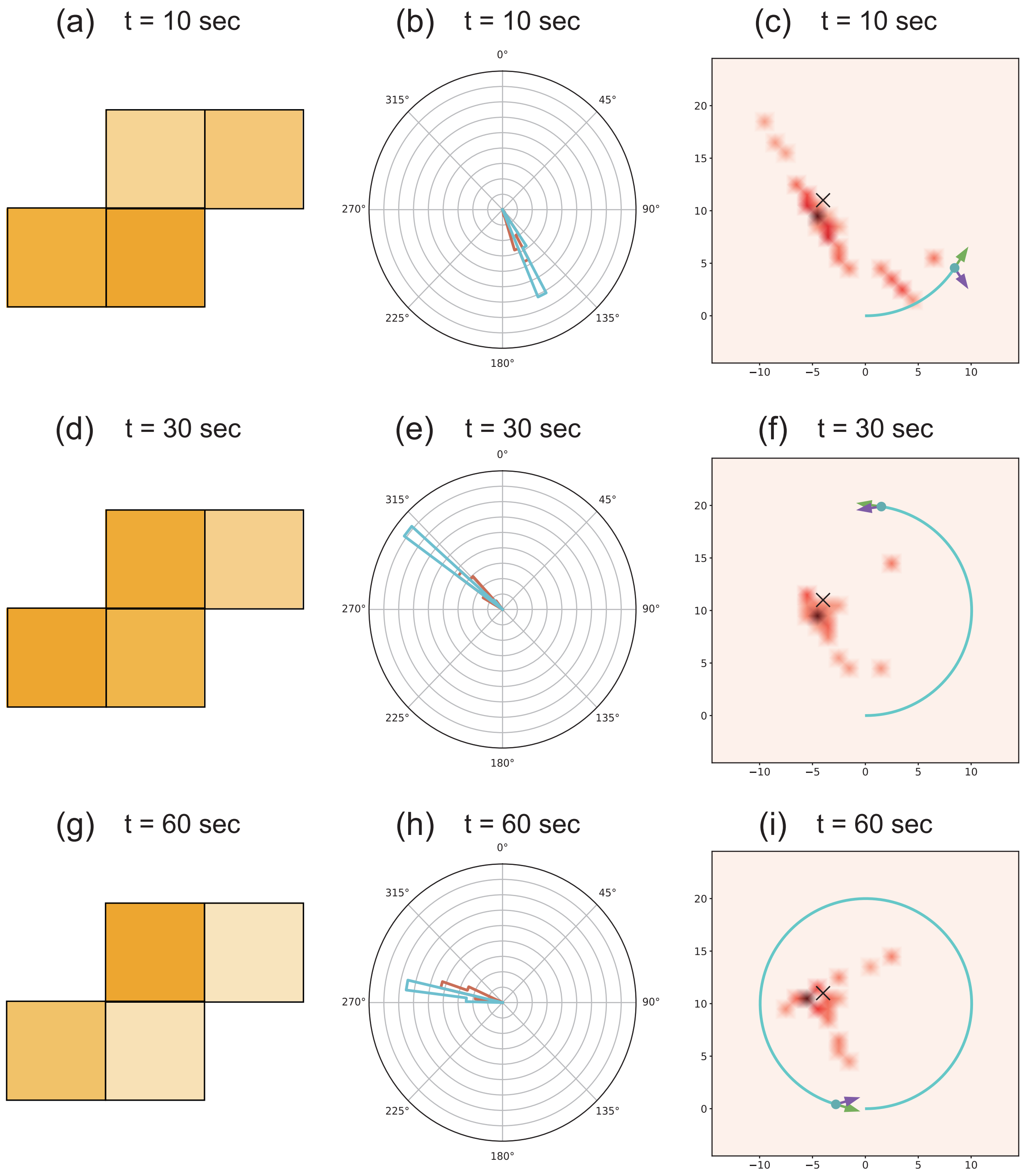

Figure S19. Radiation mapping with an S-shape Tetris-inspired detector ($\varphi = -2\theta$).
a,d,g. The detector's input signals at t=10, 30, 60 sec. The panels with intense colors represent larger values on the detector's signal. The top side of the detector represents the front side of the moving detector. **b,e,h.** The predicted direction of the radiation source at t=10, 30, 60 sec. On the polar coordinates, the blue and brown curves represent the ground truth and predicted direction of the radiation source. **c,f,i.** The process to map the radiation source at t=10, 30, 60 sec. The "×" symbols on the maps show the correct position of the radiation source. The purple and green arrows indicate the front side of the moving detector and the traveling direction, respectively. Areas with intense red colors indicate sites with a high probability of possessing radiation sources. The unit of length is the meter. Check the radiation mapping process in Supplementary Movie 10.

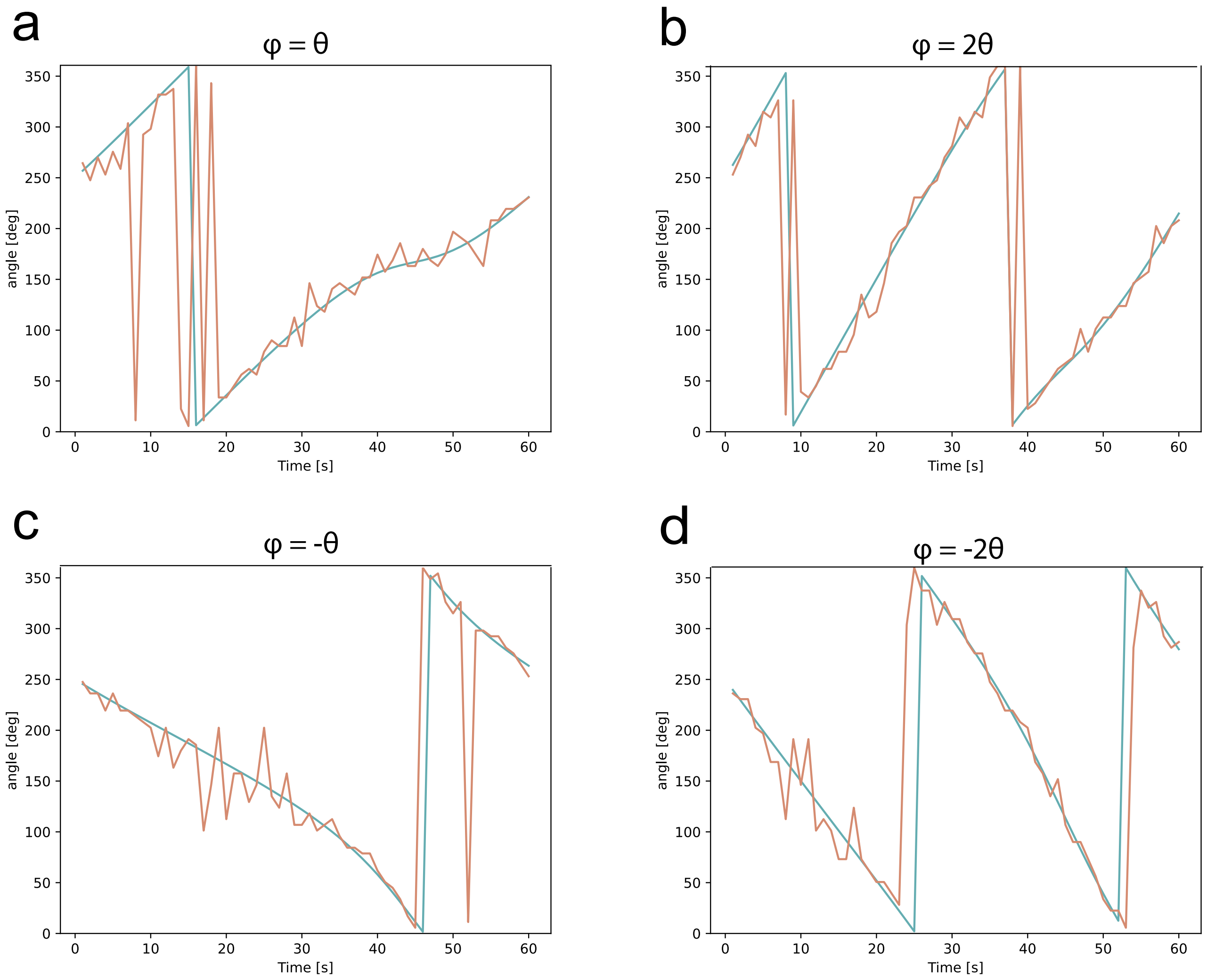

Figure S20. The predicted and actual directions of the radiation source position.
The blue and brown lines track the changes of the actual and predicted directions with detectors of **a.** $\varphi = \theta$, **b.** 2θ, **c.** $-\theta$, **d.** -2θ during the radiation mapping process of Fig. S16-S19. Here, the direction is defined as the clockwise angle from the front side of the detector.

VII Supplementary Note 7: Experimental Validation of Radiation Mapping with the 2×2 Square Detector

Figure S21 and S22 show the process of radiation mapping using experimental measurement data from two perspectives, supplying more visualization of the radiation mapping. Note that we conducted our ML analysis as a partially blinded study, i.e., the experimental team kept the radiation source location confidential until the data analysis team predicted where the actual source location was. Despite this restriction, our ML approach could locate the radiation source, verifying the performance of our radiation mapping detector in a real-world setting. Supplementary Movie 11 visualizes the process dynamically.

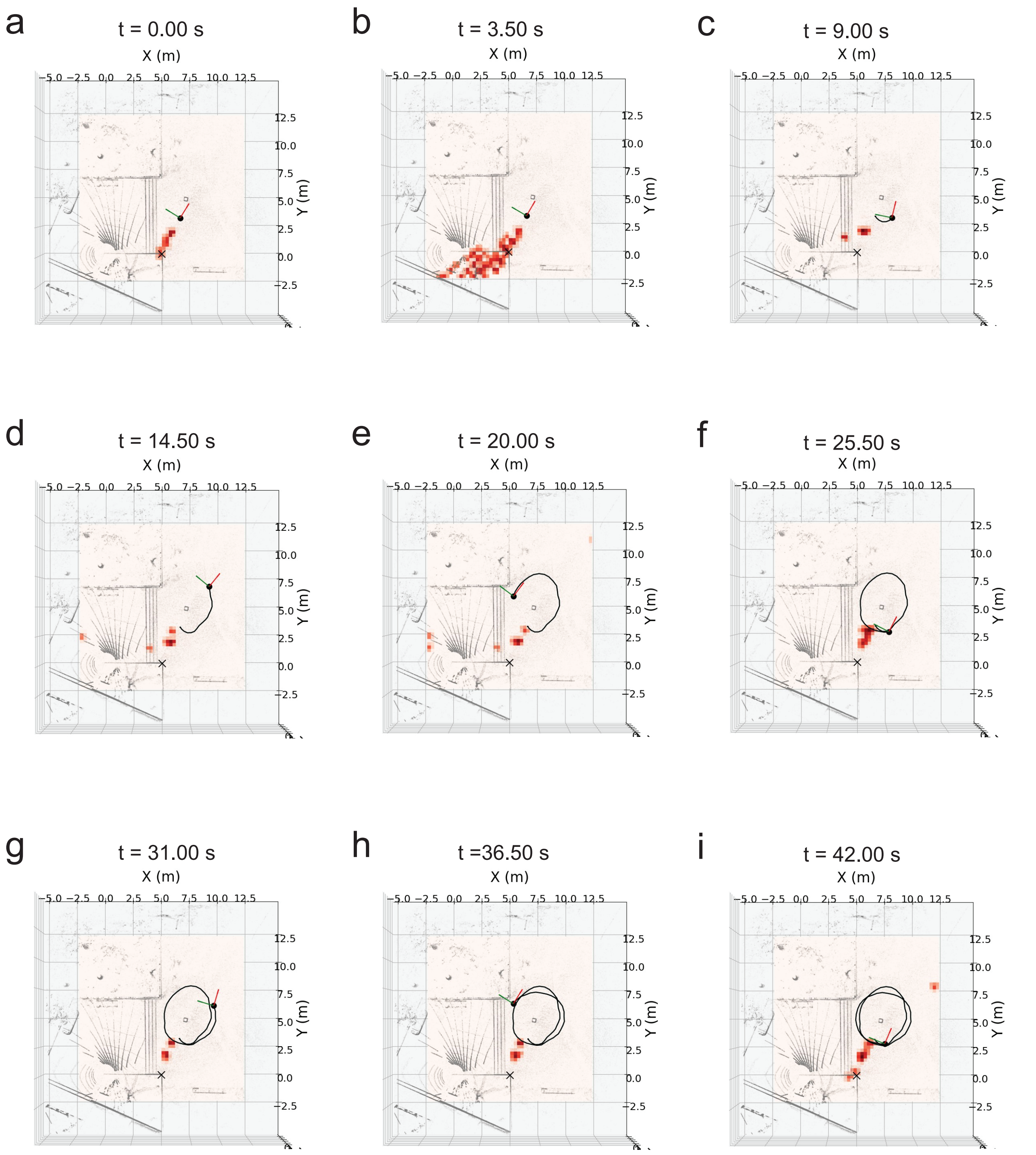

Figure S21. Mapping a radiation source location using experimental measurement data (top-down view)
We illustrate the process of mapping the radiation source location viewed from the top of the experimental space. **a-i.** The progression of radiation source mapping at representative time intervals of t = 0.0, 3.5, 9.0, 14.5, 20.0, 25.5, 31.0, 36.5, and 42.0 seconds, respectively. The black point clouds in the diagrams represent the surrounding environment of our experiment. The symbol ”×” designates the ground-truth location of the radiation source. The black dot on the maps indicates the position of the radiation detector. We visualize the detector’s left (y) and front (x) axes with green and red arrows, respectively. The black solid line indicates the trajectory of the detector. Check the radiation mapping process in Supplementary Movie 11.

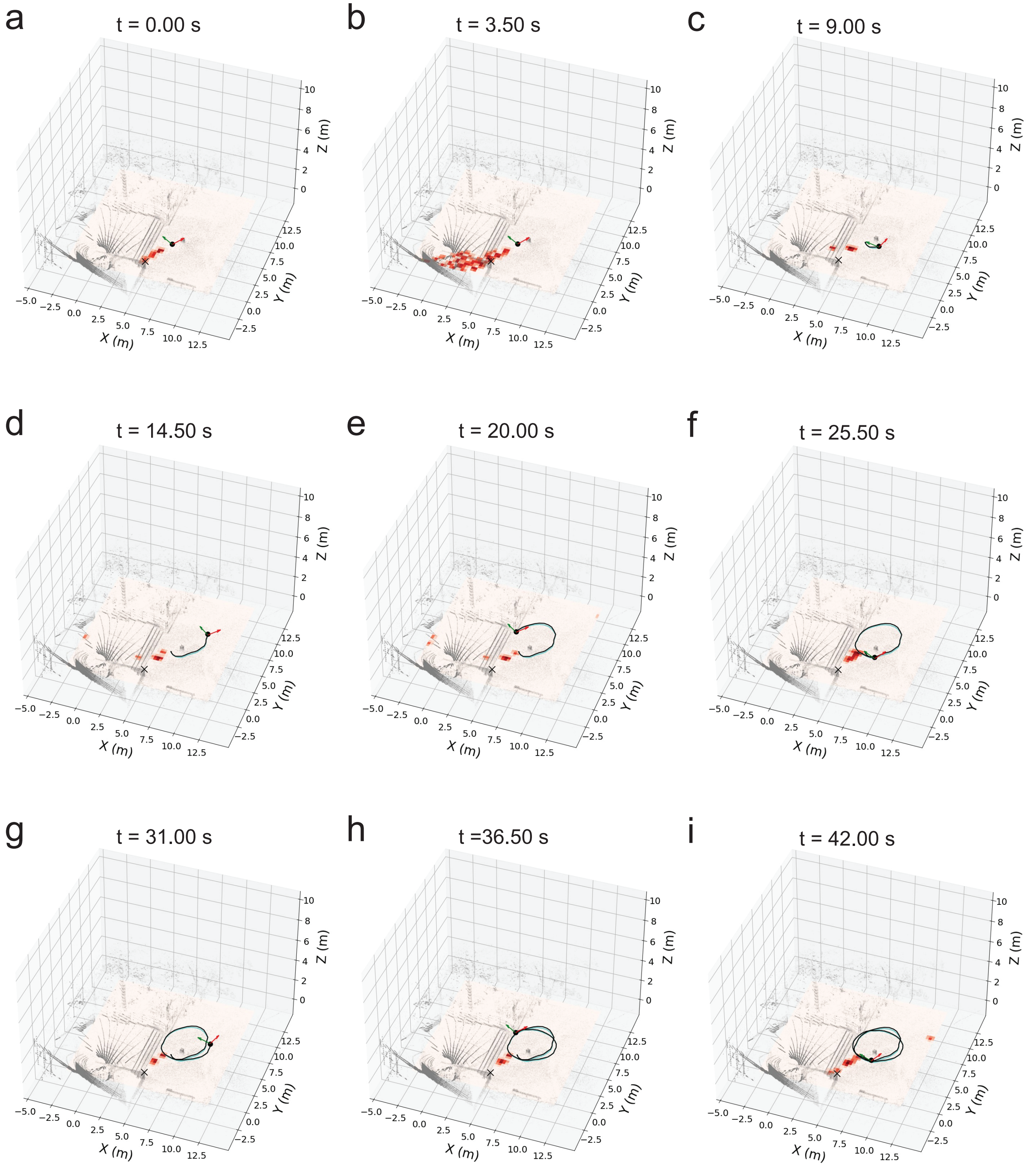

Figure S22. **Mapping a radiation source location using experimental measurement data (aerial view)**
We illustrate the process of mapping the radiation source location viewed from the aerial perspectives of the experimental space. **a-i.** The progression of radiation source mapping at representative time intervals of t = 0.0, 3.5, 9.0, 14.5, 20.0, 25.5, 31.0, 36.5, 42.0 seconds, respectively. The black point clouds in the diagrams represent the surrounding environment of our experiment. The symbol ”×” designates the ground-truth location of the radiation source. The black dot on the maps indicates the position of the radiation detector. We visualize the detector's left (y) and front (x) axes with green and red arrows, respectively. The black solid line indicates the trajectory of the detector. The blue trajectory is a projection of the actual trajectory on the xy-plane of the space. Check the radiation mapping process in Supplementary Movie 11.

VIII Supplementary Note 8: Guidance for Running the Codes for Radiation Mapping

In this section, we provide the detailed instructions for reproducing our results of simulations and machine learning. Please find our github repository for detailed codes and archived data. **(1) MC simulation to get training data**

We generate the training data for our direction prediction. Using OpenMC library, we simulate the radiation detector of square (or Tetris) configurations absorbing radiation from source(s) of random positions in specified distances. You need to set the following parameters.

- **num_sources**: The number of radiation sources to place in the field.
- **a_num** (only for the square detector): The parameter for the configuration of the detector: a x a square shape.
- **shape_name** (only for the Tetris detector): Tetris shape ['S', 'J', 'T', 'L', 'Z']
- **num_data**: The number of the generated data.
- **seg_angles**: The number of angle sectors (angular resolution: 360 deg/seg_angles).
- **dist_min**: Minimum distance between the radiation source and the detector (cm).
- **dist_max**: Maximum distance between the radiation source and the detector (cm).
- **source_energies**: Photon energy of the radiation (eV).
- **num_particles**: The number of photon in MC simulation.
- **run_name**: The folder name to save the simulation results.

```
$ python gen_data_tetris.py (or gen_data_square.py)
```

(2) MC Simulation to Get Filtering Layer

We generate the filter layers for our direction prediction. Using the OpenMC library, we simulate the radiation detector of Tetris (or square) configurations absorbing radiation from sources. You need to set the following parameters:

- **a_num** (only for the square detector): The parameter for the configuration of the detector: an $a \times a$ square shape.
- **shape_name** (only for the Tetris detector): Tetris shape $['S','J','T','L','Z']$.
- **num_data**: The number of the generated data. Use the same number as 'seg_angles' in training data generation.
- **source_energies**: Photon energy of the radiation (eV).
- **num_particles**: The number of photons in MC simulation.
- **header_dist_particles_dict**: The profile of the distance and the number of photons for each set of filter layers.
- **run_name**: The folder name to save the filter layer files.

To generate the filter layers, execute:

```
$ python gen_filter_tetris.py (or gen_filter_square.py)
```

(3) Training

We train the U-Net architecture with filter layers for predicting the directions of the radiation sources. We use the simulation data and filter layers generated above. You need to set the following parameters:

- **num_sources**: The number of radiation sources to place in the field. Set the same value as the one you used for generating training data.
- **seg_angles**: The number of the generated data. Use the same number as 'seg_angles' in training data generation.

- **epochs**: The total iterations to train the model.
- **data_name**: Training data folder name. Set it the same as the folder name you used in gen_data_tetris.py (gen_data_square.py).
- **test_ratio**: The ratio of data used as the testing dataset.
- **k_fold**: The number setting for k-fold cross-validation.
- **filter_name**: Filter layer data folder name. Set it the same as the folder name you used in gen_filter_tetris.py (gen_filter_square.py).
- **save_name**: The name for saving the model.

To train the model, execute:

```
$ python train_model.py
```

(4) Simulation with a Moving Detector

We generate input files for radiation mapping. We consider the situation where the radiation source(s) is placed at fixed position(s), and the detector moves on the trajectory to map the radiation. You need to set the following parameters:

- **input_shape**: (int) the size of the square detector (a_num), or ['J', 'L', 'S', 'T', 'Z'] (string) for the Tetris detector (shape_name).
- **seg_angles**: The number of angle sectors (angular resolution: 360°/seg_angles). Make sure to use the same value as those of training data and filters.
- **model_name**: Model name trained with the code 'train_model.py'.
- **RSID**: The position of radiation source(s) in 2D space. The shape of the array is (n, 2), where n is the number of radiation sources.
- **rot_ratio**: Rotation ratio χ, where $\phi = \chi \times \theta$.
- **DT**: The digit of time (s).
- **SIM_TIME**: The total simulation time (s).
- **SIM_STEP**: The simulation time step, each of which we save pkl files.
- **num_particles**: The number of photons in MC simulation.
- **map_horiz**: The map geometry in the horizontal axis (m). (The bottom position, the top position, the number of pixels).
- **map_vert**: The map geometry in the vertical axis (m). (The left position, the right position, the number of pixels).

To run the detector simulation, execute:

```
$ python run_detector.py
```

(5) Radiation mapping

The code produces the images of radiation mapping at each timestamp. After finishing processing the data of all time, it generates the image visualizing the mapping process. You need to set the following parameters:

- **fig_header**: The folder name header where run_detector.py saved pkl files.
- **th_level**: Threshold level of the map. We regard the map value zero at each pixel if the value after normalization is below this threshold level.
- **map_horiz**: The map geometry in the horizontal axis (m). Set the same values as 'run_detector.py'.
- **map_vert**: The map geometry in the vertical axis (m). Set the same values as 'run_detector.py'.

To generate the radiation mapping, execute:

```
$ python radiation_mapping.py
```
