# Supplementary material for: Tetris-inspired detector with neural network for radiation mapping
Source: Nat Commun. 2024 Apr 9;15:3061. doi: 10.1038/s41467-024-47338-w (PMC11004156; doi:10.1038/s41467-024-47338-w)
Supplement: Supplementary file 3 — Description of Additional Supplementary Files [file 41467_2024_47338_MOESM3_ESM.pdf]

File name: Supplementary Movie 1
Description: Radiation mapping with an S-shape Tetris-inspired detector.
(Top left) The detector's input signals over 60 seconds. The panels with intense colors represent larger values on the detector's signal. The top side of the detector represents the front side of the moving detector. (Top right) The predicted direction of the radiation source at each timestamp. On the polar coordinates, the blue and brown curves represent the ground truth and predicted direction of the radiation source. (Bottom) The process to map the radiation source. The "$\times$" symbols on the maps show the correct position of the radiation source. The purple arrows indicate the front side of the moving detector. Areas with intense red colors indicate sites with a high probability of possessing radiation sources. The unit of length is the meter.

File name: Supplementary Movie 2
Description: Radiation mapping with a 2$\times$2 square detector.
(Top left) The detector's input signals over 60 seconds. The panels with intense colors represent larger values on the detector's signal. The top side of the detector represents the front side of the moving detector. (Top right) The predicted direction of the radiation source at each timestamp. On the polar coordinates, the blue and brown curves represent the ground truth and predicted direction of the radiation source. (Bottom) The process to map the radiation source. The "$\times$" symbols on the maps show the correct position of the radiation source. The purple arrows indicate the front side of the moving detector. Areas with intense red colors indicate sites with a high probability of possessing radiation sources. The unit of length is the meter.

File name: Supplementary Movie 3
Description: Radiation mapping with a J-shape Tetris-inspired detector.
(Top left) The detector's input signals over 60 seconds. The panels with intense colors represent larger values on the detector's signal. The top side of the detector represents the front side of the moving detector. (Top right) The predicted direction of the radiation source at each timestamp. On the polar coordinates, the blue and brown curves represent the ground truth and predicted direction of the radiation source. (Bottom) The process to map the radiation source. The "$\times$" symbols on the maps show the correct position of the radiation source. The purple arrows indicate the front side of the moving detector. Areas with intense red colors indicate sites with a high probability of possessing radiation sources. The unit of length is the meter.

File name: Supplementary Movie 4
Description: Radiation mapping with a T-shape Tetris-inspired detector.
(Top left) The detector's input signals over 60 seconds. The panels with intense colors represent larger values on the detector's signal. The top side of the detector represents the front side of the moving detector. (Top right) The predicted direction of the radiation source at each

timestamp. On the polar coordinates, the blue and brown curves represent the ground truth and predicted direction of the radiation source. (Bottom) The process to map the radiation source. The "$\times$" symbols on the maps show the correct position of the radiation source. The purple arrows indicate the front side of the moving detector. Areas with intense red colors indicate sites with a high probability of possessing radiation sources. The unit of length is the meter.

File name: Supplementary Movie 5
Description: Mapping 2 radiation sources with a 10$\times$10 square detector.
(Top left) The detector's input signals over 60 seconds. The panels with intense colors represent larger values on the detector's signal. The top side of the detector represents the front side of the moving detector. (Top right) The predicted direction of the radiation source at each timestamp. On the polar coordinates, the blue and brown curves represent the ground truth and predicted direction of the radiation source. (Bottom) The process to map the radiation source. The "$\times$" symbols on the maps show the correct position of the radiation source. The purple arrows indicate the front side of the moving detector. Areas with intense red colors indicate sites with a high probability of possessing radiation sources. The unit of length is the meter.

File name: Supplementary Movie 6
Description: Mapping 2 radiation sources with a 5$\times5 square detector.
(Top left) The detector's input signals over 60 seconds. The panels with intense colors represent larger values on the detector's signal. The top side of the detector represents the front side of the moving detector. (Top right) The predicted direction of the radiation source at each timestamp. On the polar coordinates, the blue and brown curves represent the ground truth and predicted direction of the radiation source. (Bottom) The process to map the radiation source. The "$\times$" symbols on the maps show the correct position of the radiation source. The purple arrows indicate the front side of the moving detector. Areas with intense red colors indicate sites with a high probability of possessing radiation sources. The unit of length is the meter.

File name: Supplementary Movie 7
Description: Radiation mapping with an S-shape Tetris-inspired detector ($\varphi = \theta$).
(Top left) The detector's input signals over 60 seconds. The panels with intense colors represent larger values on the detector's signal. The top side of the detector represents the front side of the moving detector. (Top right) The predicted direction of the radiation source at each timestamp. On the polar coordinates, the blue and brown curves represent the ground truth and predicted direction of the radiation source. (Bottom) The process to map the radiation source. The "$\times$" symbols on the maps show the correct position of the radiation source. The purple and green arrows indicate the front side of the moving detector and the traveling

direction, respectively. Areas with intense red colors indicate sites with a high probability of possessing radiation sources. The unit of length is the meter.

File name: Supplementary Movie 8
Description: Radiation mapping with an S-shape Tetris-inspired detector ($\varphi = 2\theta$).
(Top left) The detector's input signals over 60 seconds. The panels with intense colors represent larger values on the detector's signal. The top side of the detector represents the front side of the moving detector. (Top right) The predicted direction of the radiation source at each timestamp. On the polar coordinates, the blue and brown curves represent the ground truth and predicted direction of the radiation source. (Bottom) The process to map the radiation source. The "$\times$" symbols on the maps show the correct position of the radiation source. The purple and green arrows indicate the front side of the moving detector and the traveling direction, respectively. Areas with intense red colors indicate sites with a high probability of possessing radiation sources. The unit of length is the meter.

File name: Supplementary Movie 9
Description: Radiation mapping with an S-shape Tetris-inspired detector ($\varphi = -\theta$).
(Top left) The detector's input signals over 60 seconds. The panels with intense colors represent larger values on the detector's signal. The top side of the detector represents the front side of the moving detector. (Top right) The predicted direction of the radiation source at each timestamp. On the polar coordinates, the blue and brown curves represent the ground truth and predicted direction of the radiation source. (Bottom) The process to map the radiation source. The "$\times$" symbols on the maps show the correct position of the radiation source. The purple and green arrows indicate the front side of the moving detector and the traveling direction, respectively. Areas with intense red colors indicate sites with a high probability of possessing radiation sources. The unit of length is the meter.

File name: Supplementary Movie 10
Description: Radiation mapping with an S-shape Tetris-inspired detector ($\varphi = -2\theta$).
(Top left) The detector's input signals over 60 seconds. The panels with intense colors represent larger values on the detector's signal. The top side of the detector represents the front side of the moving detector. (Top right) The predicted direction of the radiation source at each timestamp. On the polar coordinates, the blue and brown curves represent the ground truth and predicted direction of the radiation source. (Bottom) The process to map the radiation source. The "$\times$" symbols on the maps show the correct position of the radiation source. The purple and green arrows indicate the front side of the moving detector and the traveling direction, respectively. Areas with intense red colors indicate sites with a high probability of possessing radiation sources. The unit of length is the meter.

File name: Supplementary Movie 11

Description: (Top left) The detector's input signals over 42 seconds. The panels with intense colors represent larger values on the detector's signal. The top side of the detector represents the front side of the moving detector. (Top center) The predicted direction of the radiation source at each timestamp. On the polar coordinates, the brown curves represent the predicted direction of the radiation source. (Top right) We visualize the detector's coordinate space with X- and Y- axes with red and red arrows, respectively. We illustrate the process of mapping the radiation source viewed from the top-down view (bottom left) and the aerial view (bottom right) of the experimental space. The symbol "$\times$" designates the ground-truth location of the radiation source. The black dot on the maps indicates the position of the radiation detector. The red and green arrows represent the X- and Y-axes of the detector’s coordinate space, respectively. Areas with intense red colors indicate sites with a high probability of possessing radiation sources.
